# Inapparent Tick-Borne Orthoflavivirus Infection in *Macaca fascicularis*: A Model for Antiviral Drug and Vaccine Research

**DOI:** 10.3390/vaccines11121754

**Published:** 2023-11-25

**Authors:** Victoria Illarionova, Anastasia Rogova, Ksenia Tuchynskaya, Viktor Volok, Yulia Rogova, Victoria Baryshnikova, Yuriy Turchenko, Alexander Litov, Anna Kalyanova, Alexandra Siniugina, Aydar Ishmukhametov, Galina Karganova

**Affiliations:** 1 FSASI “Chumakov FSC R&D IBP RAS” (Institute of Poliomyelitis), Laboratory of Biology of Arbovirus, Moscow 108819, Russia; villarionova1@gmail.com (V.I.); rogova_aa@chumakovs.su (A.R.); kseniya-tuchka@mail.ru (K.T.); viktor.p.v@mail.ru (V.V.); rogova_jv@chumakovs.su (Y.R.); novosti-wxo@yandex.ru (A.L.); kalyanova_as@chumakovs.su (A.K.); 2 Department of Biology, Lomonosov Moscow State University, Leninskie Gory 1 bd. 3, Moscow 119991, Russia; 3 Research Institute for Systems Biology and Medicine (RISBM), Laboratory of Infectious Immunology, Moscow 117246, Russia; 4 FSASI “Chumakov FSC R&D IBP RAS” (Institute of Poliomyelitis), Laboratory of Biochemistry, Moscow 108819, Russia; baryshnikova.vs@bk.ru (V.B.); turchenko.yu@mail.ru (Y.T.); 5 Institute of Translational Medicine and Biotechnology, Sechenov First Moscow State Medical University, Moscow 119991, Russia; ishmukhametov@chumakovs.su; 6 FSASI “Chumakov FSC R&D IBP RAS” (Institute of Poliomyelitis), Moscow 108819, Russia; sinyugina@chumakovs.su

**Keywords:** orthoflaviviruses, tick-borne encephalitis virus, Powassan virus, TBE vaccine, cognitive impairment, antibody spectrum, cross-reactive antibodies, inapparent infection, flaviviruses

## Abstract

Tick-borne encephalitis virus (TBEV) and Powassan virus (POWV) are neurotropic tick-borne orthoflaviviruses. They cause mostly asymptomatic infections in hosts, but severe forms with CNS involvement can occur. Studying the early stages of viral infections in humans is challenging, and appropriate animal models are essential for understanding the factors determining the disease severity and for developing emergency prophylaxis and treatment options. In this work, we assessed the model of the early stages of TBEV and POWV mono- and co-infections in *Macaca fascicularis*. Serological, biochemical, and virological parameters were investigated to describe the infection, including its impact on animal behavior. Viremia, neutralizing antibody dynamics, and viral load in organs were chosen as the main parameters distinguishing early-stage orthoflavivirus infection. Levels of IFNα, monocyte count, and cognitive test scores were proposed as additional informative indicators. An assessment of a tick-borne encephalitis vaccine using this model showed that it provided partial protection against POWV infection in *Macaca fascicularis* without signs of antibody-dependent enhancement of infection.

## 1. Introduction

The *Orthoflavivirus* genus belongs to the *Flaviviridae* family and includes enveloped viruses with +ssRNA genome. Orthoflaviviruses are distributed throughout the world, transmitted to a wide range of animal hosts predominantly by arthropods, and can cause severe diseases of the central nervous system (CNS) or hemorrhagic syndrome depending on the tissue tropism of the virus. Orthoflaviviruses include mosquito-borne viruses: *Orthoflavivirus dengue* (dengue virus (DENV)), *Orthoflavivirus flavi* (yellow fever virus (YFV)), *Orthoflavivirus nilense* (West Nile virus (WNV)) *Orthoflavivirus japonicum* (Japanese encephalitis virus (JEV)), *Orthoflavivirus zikaense* (Zika virus (ZIKV)), etc.; and tick-borne viruses: *Orthoflavivirus encephalitidis* (tick-borne encephalitis virus (TBEV)), *Orthoflavivirus omskense* (Omsk hemorrhagic fever virus (OHFV)), *Orthoflavivirus powassanense* (Powassan virus (POWV)), *Orthoflavivirus kyasanurense* (Kyasanur forest disease virus (KFDV)), etc. [1].

TBEV and POWV are both tick-borne mammalian orthoflaviviruses belonging to the same serocomplex (i.e., having a significant antigenic overlap). Diseases caused by TBEV and POWV in humans range from inapparent and mild infections to severe fatal meningitis and encephalitis [2,3].

TBEV is widespread in Europe and Asia [2]. More than 60 million people live in TBEV-endemic territories in Russia alone, and about 12,000 cases of TBEV infection are registered annually in the European Union and Russia [4,5,6]. Based on seroprevalence studies, inapparent TBEV infections account for 70% to 95% of all TBEV infections depending on the region [7,8,9]. Acute tick-borne encephalitis (TBE) has several clinical manifestations with varying severity, including nonspecific febrile disease, meningitis, meningoencephalitis, and meningoencephalomyelitis. A biphasic course is more common for the acute TBE [10]. A significant percentage of acute cases lead to long-lasting neurological sequelae (so-called post-encephalitic syndrome) or chronic progressive TBE. Mortality for clinically confirmed TBE cases depends on several factors, including region and the virus variant, and ranges from 2% to 20% [2]. Prophylactic vaccination is the most effective measure against TBE [11,12,13,14].

POWV is widespread in Canada, the USA, and the Far East of Russia [15,16,17,18,19,20]. More than 106 cases of POWV infection have been reported in the USA from 2003 to 2018 [21]. In approximately 50% of cases, severe POWV infection leads to long-term complications, and in 10% of cases to death [22,23]. Nevertheless, the true prevalence of asymptomatic and acute POWV infections is difficult to estimate due to the lack of appropriate test systems [24]. Despite POWV being genetically related to TBEV, there are limited studies in mice on the lack of protection of TBE vaccines against lethal POWV infection [25], and there is no specific vaccine against POWV. Combined foci of POWV and TBEV exist in the Far East of Russia, so mixed infections are possible there. Previously, it has been suggested that mixed infections of POWV and TBEV could be more severe than isolated infections [26].

Factors associated with the severity of the diseases caused by TBEV and POWV are poorly understood. These factors may include genetic predisposition [27,28,29,30], dose and properties of the virus [31], immune status of the host, concomitant or prior infections, various environmental factors, and stress [2,32]. Innate immune response to flaviviruses is one of the determining factors affecting the severity of the disease, which is further underscored by a vast array of immune evasion mechanisms of flaviviruses targeting the innate response [33,34,35]. Investigation of the initial stages of flavivirus infections can provide valuable insights for understanding the factors that determine the severity of the disease course and for the development of emergency prophylaxis and treatment options. Since the tick bite is a well-verifiable event and the tick can be quickly analyzed for the presence of the virus, emergency prophylaxis seems plausible for tick-borne arboviruses, including TBEV and POWV.

The only drug for the emergency prophylaxis of TBE is a specific anti-TBE immunoglobulin, which is currently approved only in Russia [36]. Until recently, anti-TBE immunoglobulin had been used in Europe, but it has been discontinued there due to fears of possible antibody-dependent enhancement of infection (ADE) and concerns around the use of human blood products. At the moment, various classes of small molecule inhibitors of TBEV are in active development [37,38].

ADE is a phenomenon described for several orthoflaviviruses, in which non-neutralizing or low-affinity antibodies (Abs) to one virus can potentially intensify the magnitude of a secondary infection with a different virus variant or a closely related virus [39]. For mosquito-borne orthoflaviviruses, ADE has been established based on epidemiological data and demonstrated in vitro and in vivo [39,40,41]. The possibility of ADE is one of the more pressing safety issues for the development of new antiviral vaccines [42]. For tick-borne flaviviruses, the role of preexisting Abs induced during an inapparent infection is not clear, and the possibility of ADE remains open.

Initial stages of infections in humans are difficult to study because they usually happen before admission to a medical facility and, thus, are almost never observed by specialists. This is one of the reasons for the need for a valid animal model of infection. The most convenient and widespread model for studying many orthoflavivirus infections is the laboratory mouse model. For TBEV and POWV, mouse models were extensively used to study the pathogenesis, formation, and dynamics of humoral immune response, and the spectrum and cross-reactivity of antibodies induced by infection [43,44,45]. Notably, for these orthoflaviviruses, the corresponding models in susceptible mice recapitulate the pathogenesis of the severe lethal infection that is relatively rare in humans. Since mice may differ from humans in patterns of the immune response to asymptomatic infections, the data obtained using mouse models may not be entirely relevant to the human population. For example, the spectrum of DENV-induced Abs in mice is significantly different from the spectrum of human anti-DENV Abs [46,47,48].

Non-human primate models of infection, although more expensive, difficult, and ethically problematic than rodent models, are still essential for studies of pathogenesis of viral infections and antiviral immunity, especially in connection with antibody-dependent enhancement of infection (ADE). Experiments on the Old World monkeys with various routes of infection (subcutaneous, intracerebral, intraperitoneal, and intranasal) with tick-borne orthoflaviviruses showed that they are susceptible to TBEV and POWV [49,50,51,52,53,54]. The intracerebral route of infection resulted in the development of an acute disease with potential chronization; peripheral inoculation with the virus more often led to inapparent infections or to chronic infection [53,54]. It has been shown in primates that inapparent infection is capable of progressing to a chronic form, so identifying prognostic factors in inapparent infection is essential for further understanding of its progression [54].

Viremia and histological changes in the brain are well-documented signs of acute TBEV infections both in animal models and in humans [9,50,53]. Several blood biochemistry parameters were shown to reflect an ongoing acute TBEV infection in non-human primates, including increase in serum alanine aminotransferase (ALT), aspartate aminotransferase (AST), lactate dehydrogenase (LDH), and creatine kinase; decrease in serum alkaline phosphatase (ALP) and iron; and increase in ALT and LDH in the CSF [50]. However, information on inapparent infection is very limited. By analogy with other orthoflaviviral infections, several parameters could be used to characterize the inapparent infection in model animals, including levels of viremia, viral load in organs, serum cytokine profile, and blood biochemistry parameters [53,55].

One of the approaches to detect more subtle alterations in animal physiology is to observe the changes in motor activity and behavior before and after the virus infection using specialized tests. Previously, in experiments with neurotropic Semliki Forest virus, an otherwise inapparent infection in mice affected the motor activity of the animals and led to biochemical changes and virus detection in the brain before any histological changes were seen [56,57]. When wild-type mice were infected with the low-pathogenic neurotropic orthoflavivirus Langat, cognitive impairment was observed in the absence of disease signs and pathological brain changes, which was shown to be a result of hippocampal inflammation rather than direct neuronal damage [44].

There are some indications that acute infections and generalized inflammation can affect behavior and cognitive abilities. When the innate immune response is activated, pro-inflammatory cytokines such as interleukin-1α and β (IL-1α and IL-1β), tumor necrosis factor-α (TNF-α), and interleukin-6 (IL-6) can affect the cognitive functions and behavior, causing fatigue, irritability, and mild cognitive impairments (e.g., decreased attention and difficulty remembering recent events) [58,59]. These changes could possibly serve as additional markers of various infections, although there are no such data for subclinical orthoflavivirus infections in humans or primates.

In this work we attempted to model the initial stages of infection caused either by TBEV, POWV, or TBEV and POWV simultaneously (mixed infection) in monkeys *Macaca fascicularis* (also known as cynomolgus, crab-eating or long-tailed monkey). Changes in serological, biochemical, and virological parameters of study animals were evaluated to characterize the infection, and the influence of infection on animal behavior was assessed using a set of behavioral and cognitive tests. A subgroup of animals was vaccinated with an inactivated vaccine against TBEV prior to infection in order to assess how vaccine-induced Abs against TBEV would affect the experimental infection with POWV. Several animals had pre-existing Abs against orthoflaviviruses, which was taken into account along with the possibility of ADE. We assessed the cross-reactivity of Abs against TBEV and POWV at the initial stages of infection and the antibody spectrum to different domains of protein E of TBEV after vaccination and after infection with POWV. Our results indicate that TBE vaccine could potentially provide some protection against POWV in monkeys and shows no signs of ADE. Our model of inapparent orthoflavivirus infection can be further used to study the initial stages of infection and could be useful for the research and development of therapeutic and prophylactic options against orthoflavivirus infections.

## 2. Materials and Methods

### 2.1. Animals

Twelve long-tailed macaques (*Macaca fascicularis*) were used in the main challenge experiment, and additionally, 13 macaques served as controls in behavioral and cognitive tests (Table 1).

Animals were obtained from the Klenovo Veterinary Station (Klenovo settlement, Klenovo village, Centralnaya street), veterinary certificate issued by SBBZh TINAO (Animal Disease Control Station), Moscow, Novofedorovskoye settlement. The weight of macaques ranged from 1.6 to 3.5 kg. The animals were quarantined before the experiment for 1 month and housed in a specialized vivarium of the FSASI “Chumakov FSC R&D IBP RAS” (Institute of Poliomyelitis).

**Figure 1:**
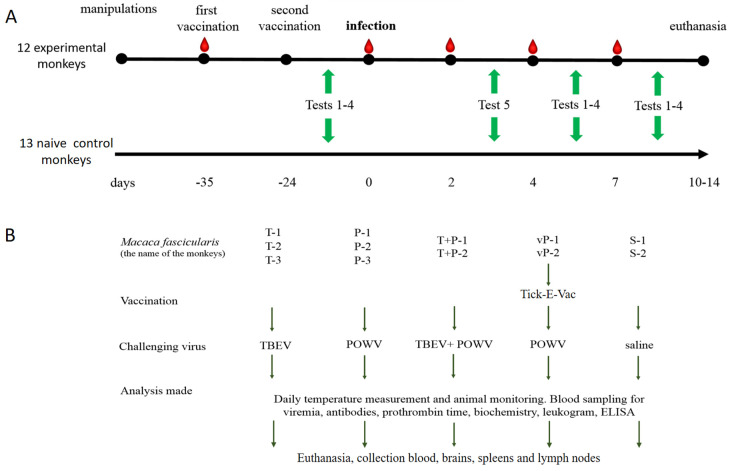
General experimental design. (**A**) Timeline of the experimental procedures. Red teardrops indicate blood sampling, green arrows indicate the periods when corresponding behavioral tests were conducted. (**B**) Description of the main experiment.

All animals were kept in accordance with CIOMS recommendations, 1985, EU Directive 2010/63/EU and Appendix A to the European Convention ETS No. 123. The study protocol was approved by the Ethics Committee of the FSASI “Chumakov FSC R&D IBP RAS” (Institute of Poliomyelitis), protocol No. 211019-2, dated 21 October 2019. All primates were housed in the same room in individual cages (100 cm × 70 cm × 80 cm) equipped with a special sliding frame for fixation of the animals. The animals were provided with a balanced diet and drinking water ad libitum and maintained in a 12 h light: 12 h dark cycle. Qualified trained personnel performed all manipulations according to the study protocol, including temperature measurements, blood sampling, injections, and behavioral/cognitive tests. The temperature was measured daily using an infrared thermometer (Sensitec NF-31-1, Apexmed, Amsterdam, The Netherlands) on the inner side of the thigh, starting 10 days before the infection. The health status of the animals was assessed daily after the virus challenge. Blood for serological and virological studies was taken from the femoral vein at —5, 2, 4, and 7 dpi (Figure 1). Euthanasia was performed at the end of the experiment at 10–14 dpi by an intramuscular injection of a mixture of anesthetic drugs zoletil (tiletamine/zolazepam, Valdepharm, Val-de-Reuil, France) and xylazine (Interchemie Werken De Adelaar Eesti AS, Puunsi, Estonia) at a dose of 0.1 mg per kg of body weight. After the injection blood was collected by a cardiac puncture. When the death of animals was confirmed by the absence of vital signs for 10 min, an autopsy and organ harvesting were performed.

### 2.2. Cells and Viruses

Porcine embryo kidney (PEK) cell line was maintained at 37 °C in medium 199 with Hanks’ balanced salt solution and Earle’s balanced salt solution (2:1, *v*:*v*, (FSASI “Chumakov FSC R&D IBP RAS” (Institute of Poliomyelitis), Moscow, Russia)), supplemented with 5% fetal bovine serum (FBS, Invitrogen, Waltham, MA, USA).

Viruses used for the challenge and the plaque reduction neutralization test (PRNT_50_) are described in Table 2.

### 2.3. Vaccination and Virus Challenge

The design of the experiment is presented in Figure 1A. Animals were divided into groups based on the random number method. In advance, two monkeys were intramuscularly (i/m) vaccinated twice with an 11 d interval with half a human dose (0.25 mL) of the inactivated vaccine against TBEV Tick-E-Vac (FSASI “Chumakov FSC R&D IBP RAS” (Institute of Poliomyelitis), Moscow, Russia). At d 24 after the second vaccination, two vaccinated and eight intact monkeys were s/c challenged with 6 log PFU of the corresponding virus. Two control monkeys were injected with sterile saline, and 13 monkeys were not subjected to any medical manipulation and were used as naive controls in cognitive experiments (Figure 1B).

### 2.4. Plaque Assay and Neutralization Test (PRNT_50_)

Plaque assay and 50% plaque reduction neutralization test (PRNT_50_) were performed in PEK cell culture in 24-well plates overlaid with 1.26% methylcellulose solution in growth medium (Sigma, St. Louis, MO, USA), as described in [60]. The infectious virus titer was expressed in log PFU/mL.

For PRNT_50_, 4-fold dilutions of sera from individual monkeys in medium 199 supplemented with 2% FBS were incubated with 30–40 PFU per well of TBEV, POWV, or WNV (1:1, *v*:*v*) for 1 h at 37 °C. Virus-serum mixture (100μL) was added to PEK cells in 24-well plates, and were then treated according to the procedure described for the plaque assay. Every experiment included negative and positive murine sera with known antibody titers as controls. The neutralizing antibody (NAb) titers were calculated according to the modified Reed and Muench method [61].

### 2.5. Measurement of IFN-α

Levels of interferon alpha (IFNα) in serum were measured using IFNα ELISA kit (Cloud-Clone Corp., Houston, TX, USA) specific for rhesus monkeys in accordance with the manufacturer’s instructions. Results were detected by absorbance measurement at 450 nm on Multiscan Microplate Spectrophotometer (Thermo Fisher Scientific, Waltham, MA, USA). Concentrations of IFNα were calculated relative to the calibration curve plotted using standard samples included in the kit.

### 2.6. Sample Preparation for Virus Detection in Organs and Blood

For viral RNA detection and virus quantification, pieces of organs and blood clots were homogenized in sterile saline solution by TissueLyser II (QIAGEN, Hilden, Germany) for 7 min at 20 Hz. Samples were stored in aliquots as 10% suspension at −70 °C before use.

### 2.7. RNA Quantification (qRT-PCR)

Total RNA was isolated from 10% organ suspensions by TRI Reagent LS (Sigma, St. Louis, MO, USA) according to the manufacturer’s instructions. A fixed amount (5.5 log RNA copies per sample) of attenuated type I Sabin poliovirus vaccine strain was added to each sample as an internal control, as described in [62]. Reverse transcription (RT) was performed with M-MLV reverse transcriptase (Promega, Madison, WI, USA), according to the manufacturer’s protocol. Sequences of oligonucleotide primers (Synthol, Moscow, Russia) are shown in Table S1.

Standards for qRT-PCR were made according to the protocol described earlier [60]. qRT-PCR was performed on C1000 Thermal Cycler with Chromo4 detector (BioRad, Hercules, CA, USA), using Taq polymerase, RealTime PCR kits, and primers (Synthol, Moscow, Russia). The limit of detection for this method was determined by serial dilutions as 100 copies per reaction tube of TBEV or POWV RNA. RNA quantities in samples were expressed as log of genome-containing particles (GCP) per ml (log GCP/mL).

The RT-qPCR protocol used in this work allowed for a simultaneous RNA quantification of TBEV and POWV in a single sample. Validation of this multiplex system is presented in Figure S1. We tested the specificity of both sets of primers for the detection of TBEV and POWV. Both systems did not amplify the corresponding heterologous virus. High amounts of spiked heterologous virus (10^5^ GCP POWV and 10^6^ GCP of TBEV) had no impact on the specificity of qPCR reactions.

### 2.8. Viremia Detection

Blood clot suspensions (10%) were passaged in PEK cells to increase possible viral titers. Each sample was added to PEK cells in duplicates and incubated for 1 h at 37 °C in 5% CO_2_. The cells were washed twice with medium 199 with Earle’s salts to remove erythrocytes, 200 µL of the same medium with 2% FBS was added to each well, and the plates were incubated for 48 h at 37 °C in 5% CO_2_. The virus in the collected cell culture fluid was quantified by three methods: plaque assay in PEK cells, qRT-PCR, and ELISA for TBEV antigen using VectoTBEV antigen kit (Vector-Best, Novosibirsk, Russia).

### 2.9. Blood Analysis

Leukocyte formula was manually calculated in dry, fixed blood smears stained with May–Grunwald’s eosin-methylene blue dye and azure-eosin dye by Romanovsky (Abris, Russia) according to the manufacturer’s instructions.

Biochemical analysis of the blood sera was carried out using an automated biochemical analyzer Cobas C111 (Roche, Basel, Switzerland) calibrated for alanine aminotransferase (ALT), aspartate aminotransferase (AST), glucose (GLU), alkaline phosphatase (ALP), total protein (TP), albumin (ALB), blood urea (BUN), bilirubin total (BILT), lactate dehydrogenase (LDH), phosphate (PHOS), calcium (CA), triglycerides (TRIGL), cholesterol (CHO), gamma-glutamyl transferase (GGT), amylase (AMYL), pancreatic amylase (AMY-P), and iron (IRON).

Prothrombin time (PT) was calculated using blood samples collected in test tubes with 3.8% tri-substituted sodium citrate (Eco-Service, Moscow, Russia) in a ratio of 9:1. PT was calculated by a nonautomatic method using Diagem-P test kit (Renam, Moscow, Russia) according to the manufacturer’s instructions.

### 2.10. ELISA Based on Recombinant Proteins

Recombinant domains of the E protein (sE, dI + II, and dIII) of the Siberian TBEV subtype strain Sukhar (GenBank #OP185392) were used as antigens for the in-house ELISA assay [63,64].

Solutions of recombinant proteins (sE, dI + II, and dIII) and PEK cell lysate as a negative control were incubated in high-binding 96-well plates in the amount of 12 ng per well at 4 °C overnight. Nonspecific binding sites were blocked for 1 h at 37 °C with 4% solution of low-fat milk in PBS (FSASI “Chumakov FSC R&D IBP RAS” (Institute of Poliomyelitis), Russia) with 0,05% Tween 20 (Sigma, St. Louis, MO, USA) (PBST), and then wells were washed with PBST. Serial dilutions of studied sera were prepared and added to the wells for 1 h at 37 °C. Then, the plates were incubated with an HRP-conjugated secondary antibody (Abcam, Waltham, MA, USA) for 1 h at 37 °C. Substrate solution of TMB (Sigma, St. Louis, MO, USA) was added to each well and incubated for 30 min at RT in the dark, and then the reaction was stopped with 2M H_2_SO_4_ solution. Results were detected by measuring absorbance at 450 nm. The serum was considered positive for anti-TBEV antibodies if the optical density (OD) of the sample was twice as high as the OD of negative control wells. A titrated positive serum was used to create a calibration curve for calculating the antibody titer of the studied sera. 

### 2.11. Behavioral and Cognitive Tests

A set of behavioral and cognitive tests was selected and adapted to detect possible impairments in memory, attention, and exploratory activity, and to assess tiredness and motivation in primates during the inapparent orthoflavivirus infection and formation of antiviral immune response. The monkeys were habituated to human proximity and trained for a total of 50 days prior to any medical interventions (blood sampling and virus challenge) and tested before the challenge and at 3, 5, and 9 dpi. The virus challenge was carried out in 10 animals, with 2 animals receiving saline as infection-free controls and 13 naive control monkeys undergoing no medical procedures. The experimental design and details are shown in Figure 1 and Table 1. Naive control monkeys were tested a few days before or after the experimental group due to the amount of time needed to test each individual. Behavioral tests and training were conducted by two specialists with experience working with primates. To avoid hostility from the primates, two separate teams of researchers performed behavioral tests and medical procedures.

#### 2.11.1. Battery of Tests #1 “Tool Use and Properties”

Description: The tasks and procedures “Tool Use and Properties” were adopted from the study by Herrmann et al. [65]. We made changes to the experimental procedure, and the materials were adjusted to be operable for long-tailed macaques. Through a series of tasks, we tested the physical cognition skills of the subject primates, which included understanding objects, their properties, and spatial and cause-and-effect relationships between objects. The tests were conducted several days before the infection and at 5 and 9 dpi. Each primate was given several attempts for each test to avoid random choice. Photo materials of the testing are given in the Figures S2–S5. Scores and information are given in Table 3.

1. Procedure: “Three Cups” Test: the experimenter placed a platform in front of the primate’s enclosure and arranged three identical cups in a row. The experimenter randomly placed a treat into one of the cups in a way that the primate could fully observe it. The test was considered successful if the subject chose the cup with the treat on the first attempt. The test lasted for 3 min. This test was necessary to train the subject in object manipulation on the platform, gradually increasing the complexity of the tasks.
2. “Tool Use: Pulling a Cloth with a Treat” Test: The experimenter set up the platform in front of the primate’s enclosure and placed a piece of cloth (30 cm × 15 cm) onto it, with an edible treat positioned on top of the cloth in a way that the primate could reach the cloth but could not reach the treat directly with its paw. The primate had to use a tool (the cloth) to obtain the treat, demonstrating the ability to manipulate objects outside the enclosure and understanding the spatial relationship between objects. The test lasted for 3 min.
3. “Tool Properties: Pulling a Thread with a Treat” Test: The experimenter placed a single thread (15 cm) on a platform, and the treat was tied to the far end of the thread, out of reach of the subject. The subject was required to use the tool (thread) to reach the treat, thereby demonstrating the ability to manipulate objects outside the enclosure. The test lasted for 3 min.
4. “Tool Properties: A Whole and a Cut Thread with Treats” Test: The experimenter set up a barrier and placed two threads (15 cm) on the platform, one of which was cut in half. Both threads were identical in length, color, and material. The treat was attached to the far end of the threads, out of reach of the primate. After removing the barrier, the subject could only retrieve the treat by pulling the intact thread, thereby demonstrating the ability to manipulate objects outside the enclosure and understanding the properties of objects. If the subject initially pulled the cut thread, the test was stopped. The test lasted for 3 min.

#### 2.11.2. Battery of Tests #2 “Memory”

Description: The “Memory” tasks and procedures were adopted from the study by Herrmann et al. [65]. We made changes to the experimental procedure, and the materials were adjusted to be operable for long-tailed macaques. Through a series of tasks, we tested the memory functions of subject animals, including their ability to memorize and manipulate information. The tests were conducted several days before the infection and on 5 and 9 dpi. Each primate was given several attempts for each test to avoid random choice. Photo materials of the testing process are presented in the Figure S6. Scores and information are given in Table 4.

Procedures

1. “Spatial Memory: 3 Upside Down Cups and 1 Treat” Test: Three identical cups were arranged upside down in a row on a platform in front of the tested subject’s cage. The experimenter then presented a treat to the subject and placed it under one of the three cups (randomly chosen), allowing the subject to fully observe the process. If the subject initially chose an empty cup, it was not allowed to make any further choices. The response was considered correct when the subject selected the cup with the treat on the first attempt, thus demonstrating spatial memory abilities and focus on the target object.
2. “Sticker Cup” Test: Three inverted cups were arranged in a row on a platform in front of the subject’s cage, with one cup marked with a white sticker. The experimenter then presented a treat to the subject and placed it under the cup with the sticker, allowing the primate to fully observe the treat. If the primate initially chose a cup without the sticker, it was not allowed to make further choices. The response was considered correct when the primate selected the cup with the sticker on the first attempt. During the training process, it was necessary to establish an association between the sticker and the treat in the subject’s mind. During the training period, subjects were given the opportunity to take the test and acquire skills over approximately 8 days at various intervals, with 3 attempts each day.
3. “Memory and Association: Sticker Cup and a Barrier” Test: The subject was offered this test only if it had successfully completed the “Sticker Cup” test. The experimenter set up a barrier in front of the tested subject’s cage that completely covered the view of the subject. Then experimenter placed three upside-down cups in a row on the platform, one of which was marked with a white sticker, and a treat was placed under the cup with the sticker. The barrier was removed, and the subject was allowed to make a choice. Based on the experience gained from previous tests, the subject was expected to form and consolidate an association between the treat and the cup with the sticker. If the subject initially chose the cup without the sticker, it was not allowed to make further choices. The response was considered correct when the subject chose the cup with the sticker on the first attempt, thus demonstrating a consolidated memory of the object.

#### 2.11.3. Test #3 “The Box”

Description: “The Box” methodology was proposed to test memory and the skills of solving intellectual tasks. The subject monkey was given a clear plastic box with an edible treat inside (known and liked by the subject) and closed with a snap–lock lid. The subject had to determine how to open the box to retrieve the treat inside. The tests were conducted several days before the infection and at 5 and 9 dpi. The subjects were given 10 min for the task. The time it took for the subject to complete the task and the positive or negative temporal dynamics were recorded. Subsequently, only those subjects who successfully completed the task during training and found a way to open the box were allowed to participate in the experiment. Prior to infection, the test was administered once with 3 attempts, considering the best result out of all attempts. After infection, one attempt was given to solve the task. The percentage difference in value shift between 0 dpi and 5 dpi, and 0 dpi and 9 dpi of the study was calculated using the following formula:

*A* = (*S* − *F*)/*S* ∗ 100% and *A* = (*S* − *N*)/*S* ∗ 100%,

where *A* is the percentage difference in value shift, *S* is the value at 0 dpi, *F* is the value at 5 dpi, and *N* is the value at 9 dpi.

Photo materials of the testing process are given in Figure S7.

#### 2.11.4. Test #4 “Reaction to a New Object”

The evaluation of exploratory activity was carried out using our modification of the methodology “Reaction to a New Object” [66]. Photo materials of the testing are given in Figure S8.

Description: A subject animal was presented with an object, the key characteristic of which was novelty to the subject. The objects used were a red plastic cylinder with a height of 6 cm and a diameter of 6.5 cm (used for tests before the infection) and a plastic blue cone with a height of 10 cm and a diameter of 6.5 cm (given in tests following the infection as a new object along with the red cylinder). The animal was given 300 s to interact with the object(s). The testing process was videotaped (Xiaomi Yi 4K Action Camera, Beijing, China). The duration and type of the contact with the object were recorded. If the contact with the object ceased, the time was noted and labeled as “left”. If the subject continued to interact with the object after the designated 300 s mark, the experimenters continued to monitor the subject until it eventually put the object aside. Research activity was assessed by measuring the following parameters:

Latency of reaction: The duration of time from the start of the test to the first contact with the object.

Activity: The sum of the time intervals of the animal’s contacts with the object.

Concentration: The average length of time during which the subject comes into contact with the object.

Only subjects that interacted with the presented object during training were included in the experiment. Prior to experimental infection, the primates underwent training, in which object A was presented to them three times (A1, A2, A3) to familiarize it and form a memory of interaction with this object. Object A was presented at 10-day intervals after the initial presentation, and then on the following day. After one month, at 3 dpi, object A was presented together with a new object B (B1), which was previously unfamiliar to the subjects. Conventions for the test are presented in Table 5.

#### 2.11.5. Test #5 “Tiredness”

Description: Each monkey was offered a series of tests to assess its cognitive abilities (described previously). The degree of engagement, interest, and tiredness were evaluated based on the number of tests attempted and successfully completed by each subject animal. The tests were offered consecutively until the subject refused to continue, lost interest, or made 3 or more consecutive errors. In such cases, the testing was stopped, and the scores were calculated. Tests were presented in the same order, with 2–3 attempts for each test. The order and nature of the tests were familiar to each subject from previous experience. Only subjects that successfully completed tasks and earned scores during the training were included in the experiment. Scores obtained on the last day of training for a given test were considered as the baseline (point 0 of the experiment). The tests were conducted several days before the challenge and at 5 and 9 dpi.

### 2.12. Statistical Analysis

The analysis of the collected data was performed by comparing two independent samples using the Mann–Whitney (U) test with Microsoft Office Excel 2010 and GraphPad Prism 9.0.0 (GraphPad Software). Statistical analysis of the “Reaction to a New Object” test data was conducted by calculating the Wilcoxon criterion (z) using Statistica 13.5 software package. This criterion was chosen because the control and study groups showed a significant difference prior to the challenge, complicating the direct comparison of their results.

## 3. Results

### 3.1. Preexisting Antibodies to Orthoflaviviruses in Intact Animals

Blood sera of study animals were analyzed for the presence of NAb to WNV, TBEV, and POWV by PRNT_50_ assay before the start of the experiment in order to check whether all monkeys were naïve to orthoflaviviruses. Since the monkeys were brought from a nursery in Sochi, Russia, they could have had contact with mosquito-borne orthoflaviviruses, especially WNV, which is endemic in the south of Russia. In two monkeys, we detected the following NAb titers: in monkey S-1 (control group)—1.84 log_10_ to WNV, and in monkey TBE-2 (TBEV group)—1.81 log_10_ to WNV and 1.86 log_10_ to TBEV. Due to the limited panel of viruses used for neutralization assay and possible cross-reactivity of antibodies to orthoflaviviruses, we cannot definitively state the virus specificity of the detected NAbs to TBEV, WNV, or other orthoflavivirus.

### 3.2. Systemic Indicators of Inflammation and Immune Response during the Infection

During the experiment, no significant changes in body temperature, prothrombin time, serum biochemistry parameters (ALT, AST, GLU, ALP, TP, ALB, BUN, BILT, LDHI, PHOS, CA, TRIGL, CHO, GGT, AMYL, AMY-P, IRON), or in the general condition of the animals were observed (Table S2, Figures S9–S14).

**Figure 2:**
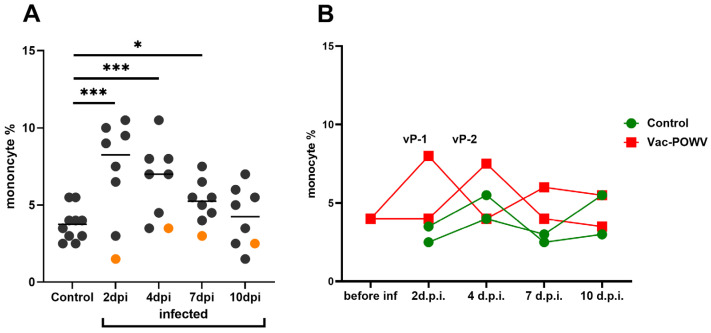
The percentage of monocytes in the peripheral blood of control and infected monkeys (**A**) The percentage of monocytes in peripheral blood at 2, 4, 7, and 10 dpi in infected group. The control group includes monocyte levels of two noninfected monkeys taken at all time points. Orange dots represent the values for the monkey TBE-2, which had pre-existing immunity to orthoflaviviruses and thus were not taken into statistical analysis. Statistical processing according to Mann–Whitney, * *p* < 0.05; *** *p* < 0.001. (**B**) Dynamics of the monocyte levels in the group vaccinated against TBEV and infected with POWV.

A significant increase in the levels of monocytes of infected nonvaccinated monkeys was observed in comparison with the control group at 2, 4, and 7 dpi. In vaccinated monkeys, relative monocytosis was observed at 2 and 4 dpi for monkeys vP-1 and vP-2, respectively, but not in monkeys with pre-existing NAbs to orthoflaviviruses (Figure 2A). This could indicate the development of the inflammatory process in response to infection. (Figure 2B). No difference in the number of lymphocytes, band, or segmented neutrophils was observed (Figure S15).

**Figure 3:**
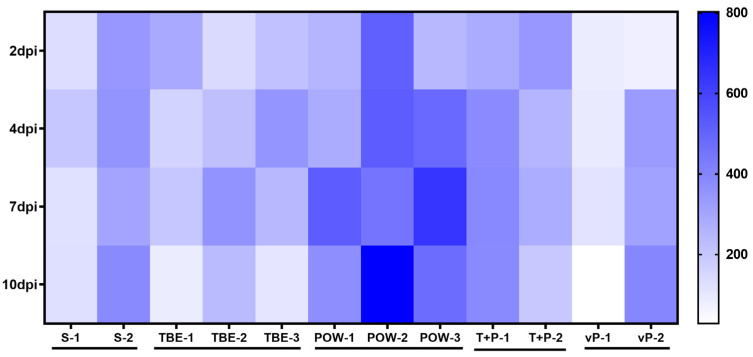
IFN-α levels (pg/mL) in the sera of infected monkeys at 2, 4, 7, and 10 dpi. Horizontal axis indicates the individual animals, vertical scale—days post-infection.

Levels of IFN-α in monkey sera are presented in a heat map (Figure 3). A small number of studied animals did not allow detecting significant differences in the levels of IFN-α. A trend towards an increase was seen for POWV-infected monkeys (POW-1, POW-2, POW-3). Among the animals infected with TBEV, mixed TBEV, and POWV, or vaccinated against TBEV and infected with POWV, no significant changes in the IFN-α levels were observed.

### 3.3. Humoral Immune Response Dynamics

At 2, 4, 6, and 10–14 dpi, monkey serum samples were taken and analyzed in PRNT_50_ assay against TBEV and POWV.

**Figure 4:**
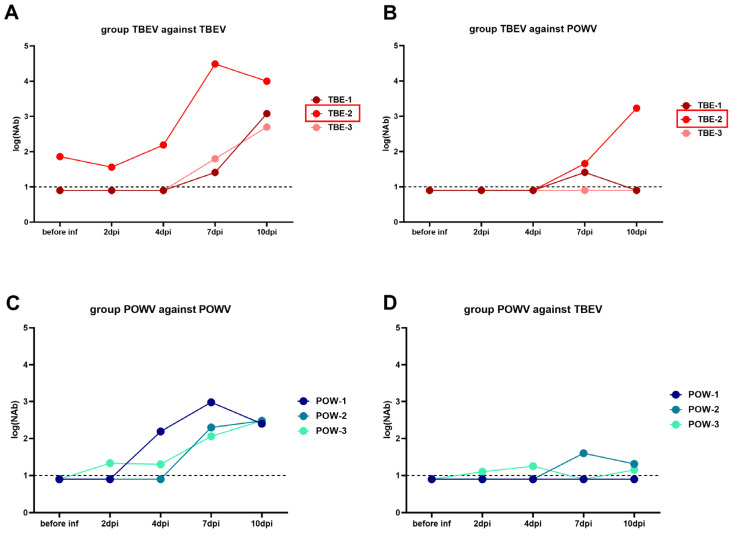
PRNT_50_ titers of NAbs in sera of orthoflavivirus-infected monkeys. Serum NAb titers against TBEV (**A**) and POWV (**B**) of TBEV-infected monkeys (TBE-1, TBE-2, and TBE-3). NAb titers against POWV (**C**) and TBEV (**D**) of POWV-infected monkeys (POW-1, POW-2, and POW-3). NAb titer values < 1 were plotted as 0.9. For anti-TBEV NAbs SD = 0.18 (measured for a positive anti-TBEV control serum, not shown), for anti-POWV NAbs SD = 0.20 (measured for a positive anti-POWV control serum, not shown). Monkeys with preexisting NAbs to orthoflaviviruses are marked with red frames. Dashed line indicates the sensitivity threshold of the assay (1 log_10_).

In the group challenged with TBEV (TBEV group), NAb titers against TBEV began to rise at 4 dpi in monkeys TBE-1 and TBE-3, and cross-reactive NAbs against POWV appeared in TBE-1 and TBE-2 at 7 dpi (Figure 4A,B). Since TBE-2 had pre-existing anti-orthoflavivirus NAbs, we observed a booster-like NAb response to TBEV at 2 dpi in this subject. No detectable cross-reactive NAbs to POWV was observed in the serum of TBE-3 at any time points.

Animals from the group infected with POWV (POWV group: monkeys POW-1, POW-2, and POW-3) had a different NAb dynamic against homological virus: for POW-3, NAb titers to POWV rose at 2 dpi, for POW-1, at 4 dpi, and for POW-2, at 7 dpi (Figure 4C). In P-3, NAb titers against POWV began to rise early at 2 dpi, suggesting that P-3 also had pre-existing immunity to orthoflaviviruses, but these NAb titers were too low to be detected in our assay before the experiment. NAb titers to heterological TBEV were low in this group and were detected in POW-3 at 2 dpi and in POW-2 at 7 dpi, and were not detected in POW-1 (Figure 4C,D).

These results indicate that cross-reactive antibodies to heterological virus can be induced after TBEV or POWV infection, which may depend on the stage of infection and the individual characteristics of the animal.

**Figure 5:**
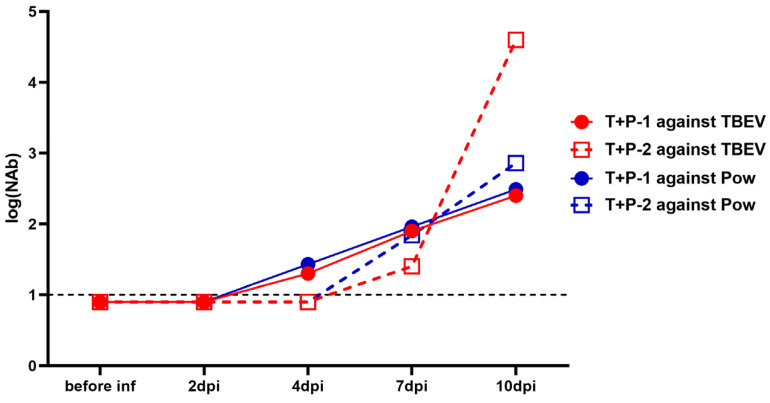
NAb titers against TBEV (red lines) and POWV (blue lines) in TBEV + POWV group. NAb titer values < 1 were plotted as 0.9. For anti-TBEV NAbs SD = 0.18 (measured for a positive anti-TBEV control serum, not shown), for anti-POWV NAbs SD = 0.20 (measured for a positive anti-POWV control serum, not shown). Dashed lines indicate the sensitivity threshold of the assay (1 log_10_).

In the group that was infected with both viruses (TBEV + POWV), the NAb dynamics were similar to the picture seen during the monoinfection. In the serum of monkey T + P − 1, NAb titers against both viruses started to rise at 4 dpi, and in T + P − 2 at 7 dpi. However, at 10 dpi, in T + P – 2, NAb titers against TBEV grew to higher levels than titers against POWV (Figure 5).

**Figure 6:**
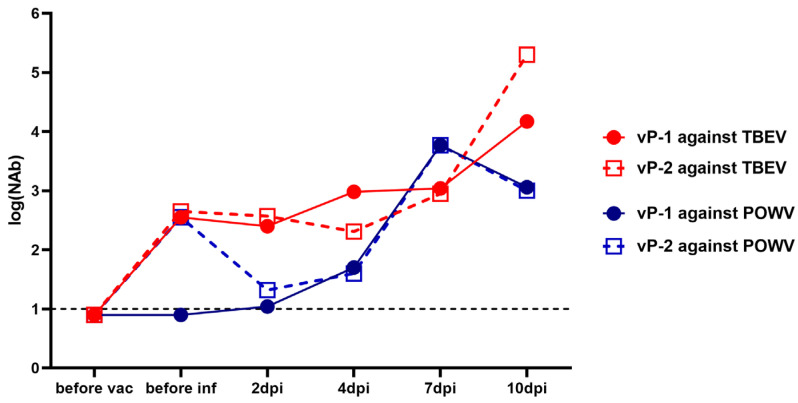
NAb titers against TBEV (red lines) and POWV (blue lines) in two monkeys vaccinated against TBEV and challenged with POWV (vP-1 and vP-2). NAb titer values < 1 were plotted as 0.9. For anti-TBEV NAbs SD = 0.18 (measured for a positive anti-TBEV control serum, not shown), for anti-POWV NAbs SD = 0.20 (measured for a positive anti-POWV control serum, not shown). A dashed line indicates the sensitivity threshold of the assay (1 log_10_).

NAb titers against TBEV in the sera of monkeys 2x vaccinated with the vaccine “Tick-E-Vac” were higher than 2 log_10_ before the challenge with POWV (Figure 6). At the same time, cross-NAbs against POWV at the same levels were detected only in the serum of one animal (vP-2). After the virus challenge, we did not detect a booster response against TBEV or POWV at 2 and 4 dpi, and NAb titers against POWV in vP-2 even decreased. This could be explained by the binding of the vaccine-induced antibodies by the actively replicating virus and their elimination from the bloodstream [60]. Four days after the challenge, there was a sharp rise in NAb titers to POWV with a peak at 7 dpi. At 10 dpi, NAb titers to TBEV continued to grow, while a small decline was observed against POWV (Figure 6). The overall dynamics of NAb titers against POWV in monkeys vaccinated against TBEV was similar to the picture seen in the absence of the vaccine.

### 3.4. Cross-Reactivity of Antibodies

**Figure 7:**
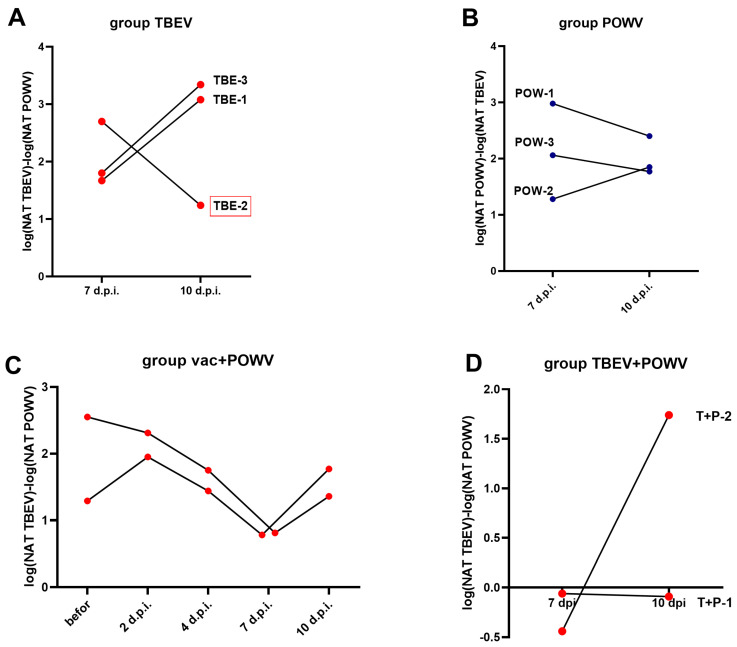
NAb ratio in TBEV- and POWV-infected groups and in vaccinated animals. (**A**) The TBEV/POWV NAb ratio in TBEV-infected group at 7 and 10 dpi. (**B**) The POWV/TBEV NAb ratio in POWV-infected group at 7 and 10 dpi. (**C**) The TBEV/POWV NAb ratio in the vaccinated group. (**D**) The TBEV/POWV NAb ratio in the TBEV and POWV infected group. Monkey with preexisting antibodies to orthoflaviviruses is marked with red frames.

We analyzed the cross-NAb titers in the monoinfected and vaccinated groups by plotting the ratio of NAb titers to TBEV against titers to POWV and vice versa (Figure 7). In the TBEV-infected group the TBEV/POWV NAb ratio increased in two animals (TBE-1 and TBE-3), and decreased in an animal with pre-existing NAbs to orthoflaviviruses (TBE-2). On the contrary, in the POWV-infected group at 10 dpi compared to 7 dpi the POWV/TBEV ratio decreased in two monkeys (POW-1 and POW-3), one of which presumably had pre-existing NAbs to orthoflaviviruses (POW-3), and only in POW-2 the POWV/TBEV NAb ratio increased at 10 dpi compared to 7 dpi (Figure 7A,B). This suggests that the NAb spectrum after infection is an individual characteristic that depends on preexisting immunity. In the group with mixed infection TBEV + POWV we observed the same TBEV/POWV NAb ratio at 7 and 10 dpi in one of the infected monkeys (T + P − 1) and an increased ratio at 10 dpi in the second monkey (T + P−2) (Figure 7D). In the vaccinated group, the TBEW/POWV NAb ratio was high at all time points, but gradually decreased at 7 dpi and began to rise again at 10 dpi (Figure 7C), which reflected the changing stages of infection.

### 3.5. Specificity of Antibodies to the Domains of the TBEV E Protein

To further investigate the antibody spectrum, we used an in-house quantitative ELISA based on recombinant TBEV proteins of the strain Suhar, which had specificity to differentiate anti-TBEV Abs from Abs to other orthoflaviviruses, including POWV [63]. As adsorbed antigens in the ELISA, we used the third domain of the E protein (EDIII), combined first and second domains (EDI + II), and a soluble part of the E protein that included all three domains (sE). Abs to all three protein variants in the sera of some monkeys with quite high Ab titers were detected (Table 6). In the TBEV-infected group, at 7 and 10 dpi, Abs were detected only in monkey TBE-2, which had preexisting NAbs. In the POWV-infected group, Abs were not detected at any time point, despite the presence of cross-reacting NAbs to TBEV. In the vaccinated group, high Ab titers to DIII, DI + II, and sE were detected at all time points except for vP-1 at 2 dpi.

**Figure 8:**
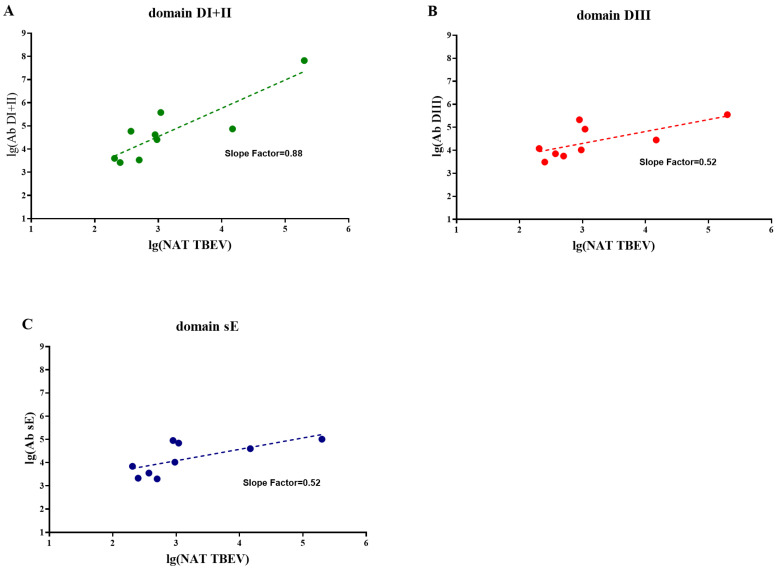
Correlation of the Ab titers to domains DI + II (**A**), DIII (**B**), and sE (**C**) of the TBEV E protein determined by ELISA and the NAbs to TBEV determined by PRNT_50_. Dashed lines indicate the trend line.

For vaccinated monkeys (vP-1 and vP-2), according to the data obtained by ELISA, we visualized the dependence of the ELISA Ab titers from NAb titers (Figure 8). The Ab titers to different domains of the E protein determined by ELISA were in direct correlation with the levels of neutralizing antibodies to TBEV. However, the slope of the trend line was the steepest for Abs to DI + II (Figure 8A). This could indicate that the greater proportion of Abs to DI + II are more non-neutralizing than among the Abs to DIII or sE.

### 3.6. Viral Load in Blood and Organs of Infected Animals

Levels of TBEV and POWV viremia in infected monkeys were assessed in blood clots, because it was previously shown that the amounts of orthoflaviviruses detected in blood clots and in serum of monkeys were equivalent [53]. Since erythrocytes could interfere with the assays, three methods were used to increase the probability of the virus detection. PEK cell culture was inoculated with 10% suspension of blood clots, and the virus presence in the cell culture fluid was studied 48 h after infection by plaque assay, TBEV- or POWV-specific qRT-PCR and ELISA for TBEV antigen detection (TBEV-Ag). The sample was considered positive if the virus was detected by at least one method. Despite the absence of visible signs of the disease, viremia was detected in all nonvaccinated monkeys at 2 dpi. Both TBEV and POWV were found in one monkey from the mixed infection group (T + P − 2) at 2 dpi. At 4 dpi, TBEV was detected in two animals from the TBEV group and again in T + P−2. In the POWV group, the virus was not detected at 4 dpi. Neither of the two monkeys vaccinated against TBEV and infected with POWV had any detectable viremia (Table 7).

The virus was not detected in the brains of infected animals in all studied groups at 10–14 dpi. However, the viral RNA was detected in the spleens and lymph nodes by qRT-PCR at 10–14 dpi in several animals. TBEV was detected in the lymph nodes of two TBEV-infected monkeys (TBE-1 and TBE-3) and in the spleen only in TBE-1. In POWV-infected group, the virus was detected in one monkey (POW-1) in the spleen and lymph nodes. In the mixed group, only TBEV was detected in the spleens of both monkeys (Table 8). In vaccinated animals no virus was detected in organs.

Consequently, a mixed POWV/TBEV infection resulted in the active reproduction of both viruses as TBEV and POWV were detected in the blood of one of the infected monkeys and cross-reactive NAbs against both viruses actively produced during infection, while only TBEV detected in the spleen of the study monkeys.

The virus was quantified by *q*RT-PCR, the number of viral RNA copies expressed in log_10_(GCP)/mL of 10% organ suspension.

### 3.7. Behavioral and Cognitive Tests Results

Considering the main limitation of this study, which is the small size of the experimental groups, in this section of the work, we combined all study animals into three different groups: infected, naive, and vaccinated monkeys. The data for each individual animal are presented on the figures and analyzed separately where appropriate. Study subjects were grouped according to a common characteristic—the presence or absence of the infection. This grouping helps to highlight the observed behavioral changes most vividly, while showing data for each animal that allow tracking the peculiarities of each individual monkey within the group and drawing accurate conclusions based on the obtained data.

#### 3.7.1. Battery of Tests #1 “Tool Use and Properties”

This set of tests was intended to assess the basic skills necessary for primates to function successfully in their natural environment. Both control subjects S (injected with saline) and C (naive control) showed improvement or maintained their previous performance at all time points during the experiment. At 5 dpi, three subjects from the virus-infected group (TBE-1, TBE-2, POW-1) exhibited a decline in performance, which was subsequently restored or surpassed at 9 dpi. Two subjects (TBE-3, POW-2) had the lowest performance levels prior to infection and demonstrated sustained improvement by 5 and 9 dpi. We did not observe a significant difference in results between the infected and control groups neither before the infection (P = 0.8215) nor at 5 dpi (P = 0.3886) and 9 dpi (P = 0.7855). The vaccinated animals were evaluated separately from the infected group and showed the following results: vP-2 exhibited a decline in performance at 5 dpi, with a positive trend observed by 9 dpi, although the scores remained below the baseline.

**Figure 9:**
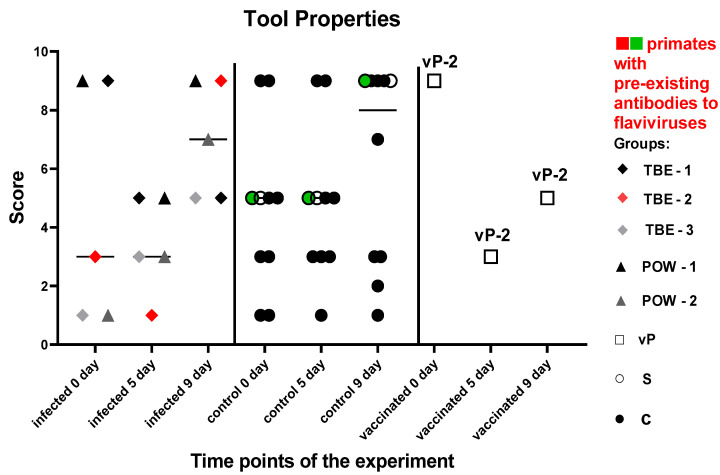
Results of the test “Tool Use and Properties” for infected and control monkeys. The statistical analysis was based on the Mann–Whitney (U) test. No significant differences were found between the infected and control groups at 5 and 9 dpi (P > 0.05). The green dots on the graph indicates the presence of pre-existing antibodies to orthoflaviviruses in the primate in the saline control group. The red dots on the graph indicates the presence of pre-existing antibodies to orthoflaviviruses in a primate from the infected group.

These results may indicate that basic cognitive functions, such as understanding object properties and ability to manipulate them, can be slightly affected during the early stages of infection in some primates (Figure 9). This effect appears to be individual-specific and temporary in nature.

#### 3.7.2. Battery of Tests #2 “Memory”

These tests were aimed at a dynamic evaluation of the state of memory and the processes of manipulation, association, and storage of acquired information in subject primates. There was no significant difference in the results between the groups before the manipulations (P = 0.2462). In the control group, all subjects except one showed an improvement or maintained the previous results. Subjects from the orthoflavivirus-infected group (TBE-1, TBE-3, POW-1) demonstrated a decrease in the results at 5 dpi, with a recovery to the baseline level by 9 dpi. The subject TBE-2 with pre-existing NAbs to orthoflaviviruses showed scores decline at 5 and 9 dpi. The infected animals had worse results at 5 dpi compared to the control group (P = 0.0434). At 9 dpi, no differences between the groups were observed (P = 0.1287), indicating a recovery of mnemonic functions and well-being in the infected animals.

**Figure 10:**
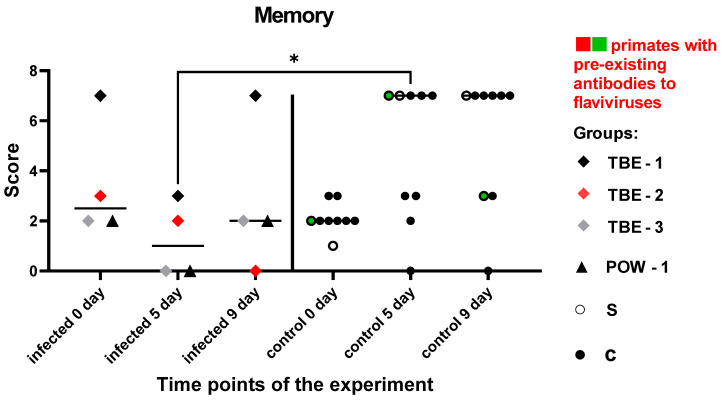
Results of the test “Memory” for infected and control monkeys. Statistical analysis was performed using the Mann–Whitney (U) test. The asterisk indicates significant differences between the infected animal group and the control group at 5 dpi (P < 0.05). The green dots on the graph indicates the presence of pre-existing antibodies to orthoflaviviruses in the primate in the saline control group. The red dots on the graph indicates the presence of pre-existing antibodies to orthoflaviviruses in a primate from the infected group.

Based on these data (Figure 10), we can hypothesize that during the orthoflavivirus infection, mild impairments in working memory and/or focus of attention may be present at around 5 dpi, with subsequent recovery of functions by 9 dpi.

#### 3.7.3. Test #3 “The Box”

**Figure 11:**
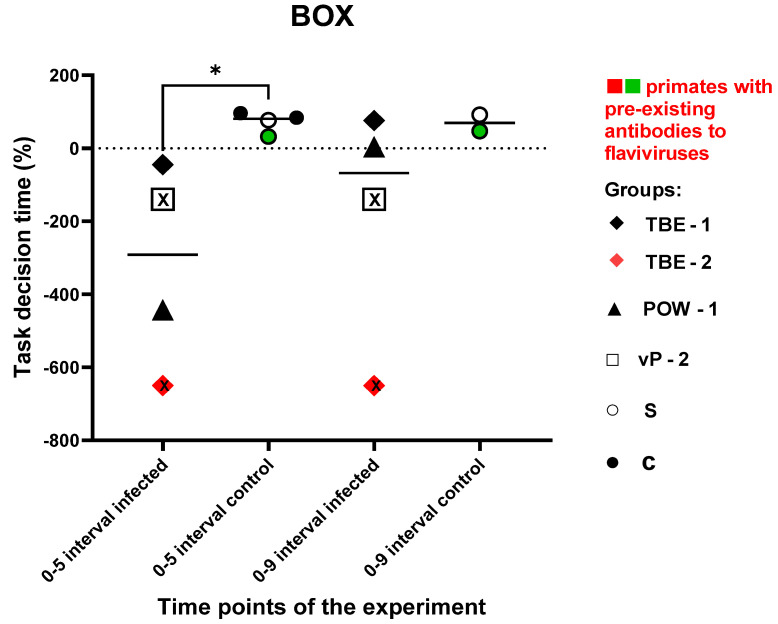
Difference in the speed of problem-solving in “The Box” cognitive task between infected and control subjects. The percentage difference in the displacement values between 0 and 5 dpi, and 0 and 9 dpi were calculated using the formula (see Materials and Methods, Test #3 “The Box”). Statistical analysis was performed using the Mann–Whitney (U) test. An asterisk indicates significant differences between the infected and the control group at 5 dpi (P < 0.05). The black “X” sign (inside the square and inside the rhombus) signifies an inability to solve the task. The green dots on the graph indicates the presence of pre-existing antibodies to orthoflaviviruses in the primate in the saline control group. The red dots on the graph indicates the presence of pre-existing antibodies to orthoflaviviruses in a primate from the infected group.

This test was aimed to assess the ability and speed of subject primates in solving intellectual tasks, as well as the memory of previously acquired skills and the dynamics of the results before and after the experimental infection. The time it took for the subject to solve the task and positive or negative temporal dynamics were recorded. Due to the small number of animals, both nonvaccinated and vaccinated subjects were included in the infected group. There was no significant difference in the speed of task solving between the experimental groups prior to all manipulations (P = 0.0857). Animals in the control group showed positive dynamics in results at 5 and 9 dpi. At 9 dpi, two subjects from the control group were not tested due to time constraints and the labor-intensive nature of the test. TBE-1 and POW-1 subjects from the infected group demonstrated a decline in performance at 5 dpi, followed by an improvement in performance at 9 dpi. TBE-2 subject with pre-existing antibodies to orthoflaviviruses showed an inability to solve the task at 5 and 9 dpi, while vP-2 subject also demonstrated the inability to solve the task at 5 and 9 dpi. Overall, the infected primates demonstrated an increase in the time required to solve the task or a complete inability to solve it at 5 dpi, when compared to the control group, which, on the contrary, showed a decrease in the time spent on solving the test (P = 0.0286). At 9 dpi, no significant differences were observed between the groups (P = 0.2667), which may have indicated the restoration of abilities and well-being in subjects from the infected group. The data are presented in Figure 11.

#### 3.7.4. Test #4 “Reaction to a New Object”

This test was carried out at 3 dpi and was intended to assess the primates’ latency of reaction, activity, and concentration, which were expressed as numerical values (parameters “Latency”, “Activity”, “Concentration”). In the baseline state, differences should be observed between the reaction to A1 (an object presented before infection) and A4 (the same object given later), indicating a decrease in interest towards a previously familiar object. The absence of a reliable difference in the response to A1 and A4 objects may indicate mnemonic impairments in subjects. Additionally, differences should be observed between the reactions to A4 and B1 (a new object presented after infection), indicating an increased interest in the new object. The absence of significant differences in the response to A4 and B1 may suggest a weak interest in novelty. The latency of reaction in the normal state is expected to be preserved or decreased (i.e., the reaction speed increases).

**Figure 12:**
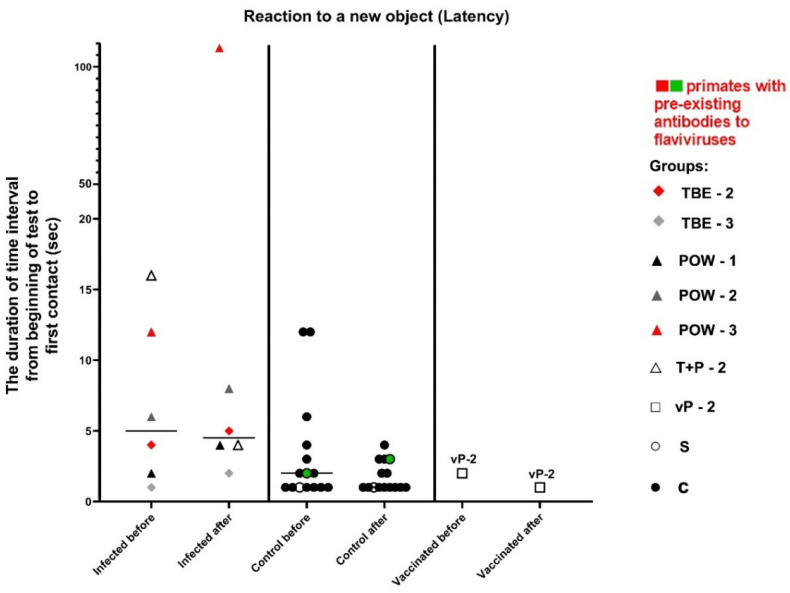
Results for the control and infected groups in the “Reaction to a New Object” test, parameter “Latency”. The vaccinated group was evaluated separately. Statistical analysis of the data was performed using the Wilcoxon (z) test. The green dots on the graph indicates the presence of pre-existing antibodies to orthoflaviviruses in the primate in the saline control group. The red dots on the graph indicates the presence of pre-existing antibodies to orthoflaviviruses in a primate from the infected group.

The parameter of latency was expressed as the duration of the time interval from the start of the test to the first contact with the presented object (A1/A4 or B1, depending on the subject’s preference when choosing between two objects). There were no statistically significant differences between the groups (Table 9, Figure 12), although an increase in reaction time was observed in almost all infected subjects.

**Figure 13:**
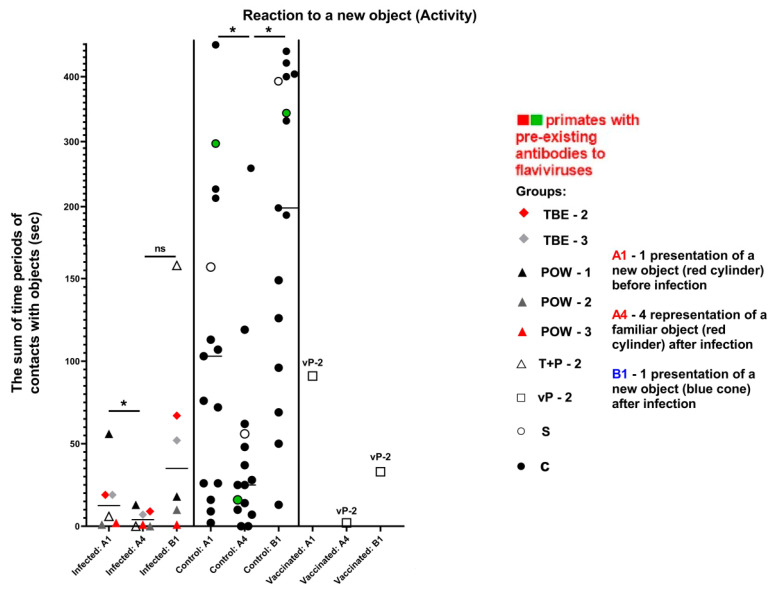
Results for the control and infected groups in the “Reaction to a New Object” test, parameter “Activity”. The vaccinated group was evaluated separately. Statistical analysis of the data was performed by the Wilcoxon (z) test. Ns—no significant difference between the previously familiar (A4) and the new object (B1) in the infected group. An asterisk indicates significant differences (z > 1.96, *P* < 0.05). The green dots on the graph indicates the presence of pre-existing antibodies to orthoflaviviruses in the primate in the saline control group. The red dots on the graph indicates the presence of pre-existing antibodies to orthoflaviviruses in a primate from the infected group.

The activity of subject animals was expressed as the sum of the time intervals of interactions between the subject and the objects. All differences in the activity of the control group were significant (Wilcoxon (z) test), indicating a healthy preference for novelty (A4/B1) and preserved memory of the previously familiar object (A1/A4). In the infected group, the results for the familiar object were significant (z = 2.2013, *P* = 0.0277), suggesting a normal differentiation between A1 and A4 and a preserved memory of the object. The results for the novel objects (A4/B1) were not significant (z = 1.1531, *P* = 0.2488), indicating no difference in the perception between A4 and B1, which may indicate a weakened interest in the new object (Table 9, Figure 13).

**Figure 14:**
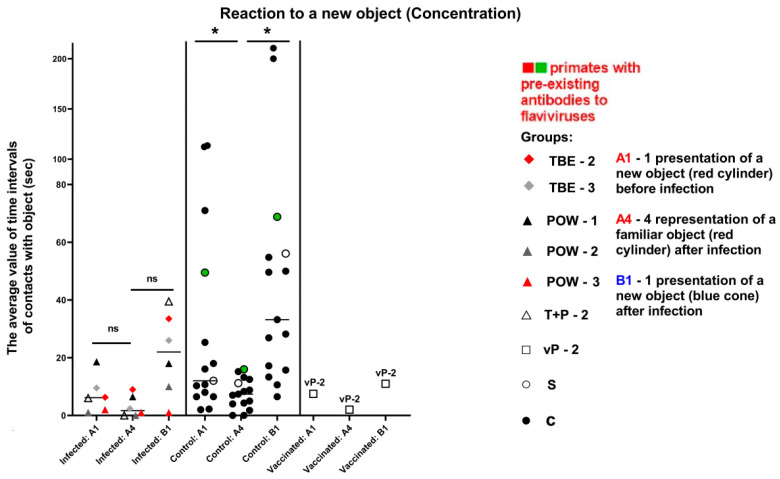
Results for the control and infected groups in the “Reaction to a New Object” test, parameter “Concentration”. Statistical analysis of the data was performed by the Wilcoxon (z) test. Ns—no significant difference in the infected group between the first encounter with the new object before infection (A1) and its subsequent presentation at 3 dpi (A4), as well as no significant difference between A1 and B1 (new object). An asterisk indicates significant differences (z > 1.96, *P* < 0.05). The vaccinated group was evaluated separately. The green dots on the graph indicates the presence of pre-existing antibodies to orthoflaviviruses in the primate in the saline control group. The red dots on the graph indicates the presence of pre-existing antibodies to orthoflaviviruses in a primate from the infected group.

The parameter of concentration was expressed as the mean length of the subject–object contacts. The control subjects showed a healthy preference for the new object, and their concentration remained stable (indicative difference between A1/A4 and A4/B1). In infected animals, we observed mild mnemonic impairments or decreased retention of attention, reflected in the small difference between A1/A4, as well as weakened interest and decreased retention of attention on the new object (A4/B1), especially in the group infected with POWV (Table 9, Figure 14).

The vaccinated subject vP-2 showed a healthy increase in the reaction speed, indicating its well-being (Figure 12). Considering the activity and concentration, vP-2 also demonstrated differences between A1/A4 and between A4/B1, indicating a preserved memory of the previously familiar object and a healthy interest and preference for the new object (Figure 13 and Figure 14).

#### 3.7.5. Test #5 “Tiredness”

This test was used to assess the degree of tiredness of test subjects, their overall psychophysiological condition, and their motivation dynamics before and after the infection. There was no significant difference in the number of successfully solved tasks between the experimental groups before the manipulations (P = 0.5752). Five subjects from the control group improved their performance at all time points of the experiment, and another five control subjects improved their results at 5 dpi and performed slightly worse at 9 dpi, which still was better than the baseline (before the infection). Two subjects from the control group decreased their performance at 5 dpi and significantly improved their results by 9 dpi. The infected group demonstrated a decrease in the number of correctly completed cognitive tasks at 5 dpi compared to the controls (P = 0.0110). Then, at 9 dpi, all subjects except (T + R) − 1 showed improvement, but their performance in average was lower than the baseline. At 9 dpi, no differences were observed between the groups, although a trend towards lower scores for the infected group could be seen (P = 0.0853). Two vaccinated subjects were evaluated separately from the infected group: vP-2 improved its performance at 5 dpi and relatively deteriorated by 9 dpi, but the results were still higher than the baseline. vP-1 refused to participate at 5 dpi and decreased its performance at 9 dpi compared to the baseline.

**Figure 15:**
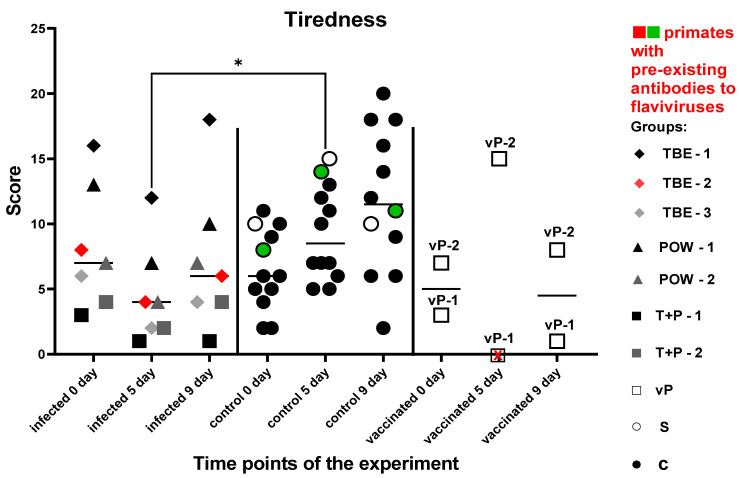
Numbers of successfully solved tasks for infected and control groups at different time points in the “Tiredness” test. The vaccinated subjects were evaluated separately. Statistical analysis was performed using the Mann–Whitney (U) test. The asterisk indicates significant differences between the infected and control groups at 5 dpi (P < 0.05). All subjects from the infected group showed a decrease in their performance at 5 dpi. The red “X” sign (inside the square) denotes the refusal of vP-1 to participate at 5 dpi. The green dots on the graph indicates the presence of pre-existing antibodies to orthoflaviviruses in the primate in the saline control group. The red dots on the graph indicates the presence of pre-existing antibodies to orthoflaviviruses in a primate from the infected group.

These data (Figure 15) may point towards reduced motivation and increased tiredness in infected monkeys at all study time points.

## 4. Discussion

The aim of this work was to investigate the possibilities and limitations of using *Macaca fascicularis* monkeys to model the initial stages of the infection caused by TBEV and POWV. Since most cases of orthoflavivirus infections in humans are asymptomatic, a primate model able to recapitulate this mild or completely inapparent infection could help to better understand the specifics of the formation of the immune response to orthoflaviviruses, and to select the most informative and unambiguous criteria for assessing the course of the clinically inapparent infection. This model or its modifications could serve as a valuable tool in the research and development of the emergency prophylaxis and novel vaccines and therapies against tick-borne neurotropic orthoflaviviruses. The main challenges of this work stemmed from the hidden nature of the initial stages of TBEV and POWV infections in macaques and from significant variations in the individual characteristics of the host. These circumstances led us to look for the widest possible range of parameters to evaluate in order to detect any changes occurring to the host organism during infection.

Acute infections with a range of tick-borne orthoflavivirus species, including TBEV, POWV, and OHFV, have been observed in non-human primates mainly after the i/c inoculation, whereas the s/c route of infection did not lead to any observable signs of the disease [49,50,51,52,53,54]. Only a single paper by Suss et al. described a clinically apparent TBE infection in monkeys after a tick bite [52]. A long-term persistence of TBEV in the brain of the monkey after the s/c challenge has also been reported, suggesting that in some cases, an inapparent TBEV infection may progress into a chronic disease [54].

In our study, we used *Macaca fascicularis* monkeys and chose the s/c route of infection as the closest to the natural way of infection by the tick bite. Our experimental design included various combinations of TBEV and POWV, including mono- and mixed infections with both viruses, and POWV infection after the double vaccination with TBE vaccine. This choice was dictated by the fact that TBEV and POWV have combined foci on the territory of the Russian Federation, and simultaneous (mixed) infections with both viruses are possible. Infection with POWV of a person vaccinated against TBE is also possible. This possibility deserves particular attention due to the risk of ADE by a mechanism described for some mosquito-borne orthoflaviviruses, like DENV. As an unexpected confounding factor, we discovered that two of the supposedly naive monkeys had preexisting anti-WNV and anti-TBEV NAbs in low titers. We could not confirm the specificity of these NAbs due to the possible cross-reactivity and limitations of the neutralization assay.

As described earlier, viremia in macaques after the s/c infection with TBEV and OHFV is usually observed between 2 and 7 dpi, while the virus can be detected in organs and the CNS at 8–10 dpi and is no longer detectable in the CNS by 20 dpi [53]. However, in another study, the presence of the virus in the brain of s/c TBEV-infected monkeys was detectable up to 350 days after infection [54]. In our study, for TBEV and TBEV-POWV mixed infections we detected viremia at 2–4 dpi, and for POWV infection only at 2 dpi. The viremia and viral load in organs clearly indicated that the infection had occurred. The virus was not detected in the brain of macaques by 10–14 dpi, a standard time point to assess the recent infection, which, together with the absence of clinical signs of the disease suggested that we have successfully modelled inapparent orthoflavivirus infection. Intriguingly, macaques vaccinated against TBE and infected with POWV showed no viremia at 2 or 4 dpi and did not have detectable virus in the organs at 10–14 dpi. This suggests that the TBE vaccine can provide partial protection against POWV infection. Considering that the number of animals having received the vaccine was too small to draw robust conclusions, further research is needed to support this potentially valuable observation.

In humans with acute TBE, the numbers of blood lymphocytes, neutrophils, monocytes, and platelets were shown to decrease in the first phase of the disease and normalize or increase during the second phase [67,68,69]. There are no similar data for inapparent tick-borne orthoflavivirus infections in humans or in primates. In monkeys infected with OHFV, a virus closely related to TBEV, leukopenia and thrombocytopenia were detected at 8 dpi [53]. In our work, we found an increase in monocyte counts in animals with mono- and mixed infection except for the monkeys with preexisting antibodies (Figure 8).

Increased levels of liver enzymes are characteristic of the initial stages of the acute TBE, with serum AST and ALT increasing in 40–60% of cases, while during the second phase of the disease, the levels of both enzymes tend to decrease [67]. An increase in AST and a normal ALT level were seen during the first days of the OHFV infection in monkeys [53]. We observed some fluctuations in the levels of ALT and AST in the sera of infected animals, but all the values were within the normal limits (described for *Macaca fascicularis* in [70]) and did not differ from the controls. We assume that these parameters could depend both on the stage and severity of infection and on the individual characteristics of the host, which complicates their interpretation and limits their usefulness. No other biochemical parameters differed significantly between the virus-infected and control animals (Table S2, Figures S9–S14).

The presence of type I IFN in the blood serum is an important feature of the activated innate immune response. In humans, increased serum IFNα has been detected during the acute TBE [67,71]. However, almost no data are available on the levels of IFNα in humans and monkeys during the inapparent orthoflavivirus infections. We measured the levels of IFNα in the sera of study animals at 2, 4, 7, and 10 dpi. The levels of IFNα increased only in POWV-infected subjects. It is known that different viruses and strains of the same virus vary in both sensitivity to IFNα and the magnitude of its induction [31]. This suggests that the TBEV strain EK-328 used for the challenge may better suppress IFNα induction than the tested POWV strain. Alternatively, these results may be explained by different dynamics of the viral reproduction of the studied viruses and the timing of their appearance in the serum.

To characterize the humoral immune response to the inapparent orthoflavivirus infection we measured the levels of NAbs against both TBEV and POWV at different time points after the infection. For monoinfections, NAbs against the homologous virus were detected in the POWV-infected group earlier than in the TBEV-infected group, coinciding with the earlier escape of the virus from the bloodstream.

In two monkeys with pre-existing NAbs against some orthoflavivirus, a booster response was observed after the monoinfection with TBEV and POWV, with NAb levels growing from 2 dpi, which was too early for a primary infection. It should be noted that this early activation of cross-reactive antiviral NAb production did not affect the viremia, i.e., did not hinder the active replication of the virus.

It is known that orthoflaviviruses exhibit serological cross-reactivity [72,73]. We assessed the degree of cross-reactivity between Abs to TBEV and POWV by calculating the ratio between NAb titers against the homologous and the heterologous viruses. In summary, NAb spectrum and cross-reactivity varied among individual animals during the monoinfections.

Before the infection in the TBE-vaccinated monkeys, cross-NAbs against POWV were detected only in one animal that had high titers of the vaccine-induced anti-TBEV NAbs. During the POWV replication in TBE-vaccinated monkeys, the induction of cross-reactive NAbs neutralizing both TBEV and POWV was observed, while the increase in anti-POWV NAbs was similar to the dynamics seen during a monoinfection. By 14 dpi, after a transient increase in anti-POWV NAbs at 7 dpi, we observed a shift in the antibody spectrum towards NAbs against TBEV. It seems that in the neutralization assays, it is not always possible to determine which orthoflavivirus caused the disease if there had been a previous exposure to other orthoflavivirus antigens in the form of a virus or a vaccine.

Neutralizing epitopes are known to be present in all domains of the protein E of TBEV, but most are located in the DIII domain, which is species-specific, while the DI + II domain is more conserved and, thus, is cross-reactive [74,75]. Previously, immune sera from humans with acute TBE were shown to contain high levels of Abs to DI + II domain [76]. In our work, we showed the presence of Abs to DIII and DI + II domains of the E protein in the sera of TBEV-infected monkeys and TBE-vaccinated animals infected with POWV, indicating some similarity in the antibody spectrum of humans and macaques. The spectrum and dynamics of NAb formation following POWV infection in the presence of pre-existing vaccine-induced Abs against TBEV were investigated for the first time. We showed that in vaccinated monkeys at the beginning of the POWV infection, there was a more pronounced increase in Abs to DI + II than to DIII, along with the rise of anti-TBEV NAbs. It is noteworthy that the slope of the linear dependence between NAb levels and the total amount of antibodies are the same for DIII and sE, and differ from that to DI + II, for which the proportion of non-neutralizing antibodies is higher.

The studies of the effects of TBEV and POWV infection on the higher neural functions of human patients revealed significant alterations in the mental status and behavior [58,77,78]. Likewise, severe impairments of the CNS have been described in primates during acute orthoflavivirus infections [79]. In our experiment, due to the inapparent character of the modeled infection, we did not anticipate any immediately obvious behavioral and cognitive changes. Nevertheless, we were able to trace the dynamics of changes in the behavior and cognitive abilities of primates.

At 3 dpi, in the “Reaction to a New Object” test, we observed a significant decrease in the interest in new objects and slight memory impairments among the infected monkeys. In the context of viremia, this effect was particularly pronounced in the group infected with POWV, where a more active increase in NAb and IFNα levels was also observed the following day. There was a trend towards increasing reaction time in the latency test in most infected subjects.

At 5 dpi, during the active antibody synthesis in the infected group, we evaluated cognitive performance of subjects. In the “Tool Use and Properties” test, no significant differences were found, but infected subjects showed slightly reduced performance, suggesting difficulties in concentrating on the task, impaired understanding of the properties of objects and object manipulation. Only two subjects with pre-existing Abs completed the task. In the “Memory” test, infected subjects had lower performance at 5 dpi compared to the controls. “The Box” test showed an increase in task-solving time in the infected group. In the “Tiredness” test, infected monkeys displayed reduced motivation and quicker became tired of the task.

At 9 dpi, despite the virus being detected at 10–14 dpi in the lymph nodes and spleen of some subjects from the infected group, and elevated serum IFNα in POWV-infected subjects, most animals showed improvement or recovery of cognitive functions compared to 5 dpi. However, in the “Tiredness” test a tendency towards lower scores was observed in the infected group, indicating increased tiredness, reduced performance, and motivation.

One vaccinated subject (vP-2) displayed positive signs at 3 dpi, including a healthy level of novelty preference, retained memory, and improved reaction speed in “Reaction to a New Object” test, indicating overall wellbeing and supporting the absence of viremia. At 5 dpi, vP-2 struggled in the “Tool Use and Properties” and “The Box” tests but performed better in the “Tiredness” test. By 9 dpi, the subject improved the results of the “Tool Use and Properties” test but still struggled in “The Box,” with lower tiredness scores than at 5 dpi. The vaccinated subject vP-1 initially refused the “Tiredness” test at 5 dpi but improved the scores at 9 dpi. Transient worsening of the cognitive test results may be associated with the active synthesis of Abs to POWV and a corresponding rapid increase in NAb titers at 4 dpi.

It is not surprising that the animals lacking antiviral Abs before the challenge had different patterns of immune response in comparison with the monkeys having post-infection and post-vaccination immunity to the heterologous viruses. Still, we can conclude that these differences in the immune response did not lead to significantly different results in a panel of cognitive and behavioral tests. In addition, behavioral tests did not reveal significant differences between monkeys infected with TBEV and POWV, or having a mixed infection. Taken together, this suggests that the observed changes were more likely related to the formation of the immune response rather than to the possible direct effects of the virus replication.

Using the battery of cognitive and behavioral tests adapted for long-tailed macaques, we were able to detect deviations in the behavior and cognitive abilities of primates at the early stage of the inapparent viral infection. Behavioral tests can serve as additional markers of the overall condition in animal models of various clinical forms of viral infections, which is particularly important for the viruses able to establish chronic infections. Behavioral tests can also be useful in assessing the efficacy of antiviral vaccines.

Our work has several limitations imposed, among other things, by the elusive and complex nature of the studied infection. The main challenges included the small size of the experimental groups, a limited amount of cytokines measured in serum, and difficulties in interpretation. Still, this work can serve as an important step in this direction of research. The presence of inapparent infection was unambiguously shown by the combination of primary distinguishing parameters, namely viremia, serum NAb dynamics, and viral load in the internal organs at 10–14 dpi. In addition, several secondary parameters that may manifest in a more pronounced way, depending on the experimental conditions, were found. They included IFNα levels, monocyte count, and cognitive test scores. A more comprehensive study on the formation of the immune response during infections caused by neurotropic orthoflaviviruses and studies of other viral infections with bigger group sizes would allow us to draw further conclusions. Additional studies could detect more markers and validate this research by the actual testing of the novel antiviral vaccines and therapeutics.

In summary, we modeled the inapparent orthoflavivirus infection in non-human primates and identified several biomarkers and time points for their assessment. This information can be used to characterize the infection in detail, including cases of prolonged viral persistence, as well as to study the efficacy of antiviral drugs and vaccines. The primary markers of the ongoing infection include viremia, rise in NAb levels, and the presence of the virus in the organs at 10–14 dpi. We have identified additional informative parameters that tended to change during the modeled infection. Changes in the serum IFN-α, monocyte counts, and alterations of the cognitive test scores can be useful and informative. Combining these parameters, we obtained preliminary data indicating that the vaccine against TBE can provide partial protection of *Macaca fascicularis* against the challenge of the Powassan virus.

## Figures and Tables

**Table 1:** Study animals.

Animals ^1^	Sex	Age, y	Intervention	Behavioral and Cognitive Tests					Euthanasia, dpi
				Battery of Tests 1 «Tool Use and Properties»	Battery of Tests 2 «Memory»	Test 3 «Box»	Test 4 «Reaction to a New Object»	Test 5 «Tiredness»	
TBE-1	F	3	TBEV	+	+	+		+	14
TBE-2	F	3		+	+	+	+	+	11
TBE-3	F	4		+	+		+	+	10
POW-1	M	3	POWV	+	+	+	+	+	11
POW-2	F	3		+			+	+	10
POW-3	F	3					+		14
T + P − 1	F	3	TBEV +POWV					+	11
T + P − 2	M	3					+	+	14
vP-1	F	3	Vac +POWV					+	14
vP-2	F	3		+		+	+	+	11
S-1	F	3	saline	+	+	+	+	+	10
S-2	M	4		+	+	+	+	+	14
C1-13	M/F	3–4	naive control	N = 8	N = 7	N = 2	N = 13	N = 10	

^1^ Abbreviations for individual monkeys are TBE—infected with TBEV, POW—infected with POWV, T + P—mixed TBEV/POWV infection, vP—vaccinated against TBEV and infected with POWV, S—injected with saline, C—naive control. Naive animals were not subjected to any manipulations and/or interventions and were used only to assess cognitive abilities. All animals were housed in the same room. dpi—days post-infection.

**Table 2:** Viruses in the experiment.

Virus	Strain	Region and Year of Isolation	Isolate Source	GenBank Accession Number	Passage History *
TBEV, Siberian subtype	EK-328	Estonia, 1972	*I. persulcatus*	DQ486861.1	M6C1M6C1
POWV	Pow-24	Primorsky Krai, Russia, 1975	*I. persulcatus*	MG652438.1	M_4_V_1_C_1_
WNV	Hp-94	Astrakhan, Russia, 1963	*H. marginatum*	JX041633.1	MxC_1-2_

* M—passage in the brain of a suckling mouse (Mx—unidentified number of early passages before the virus was obtained by the laboratory), V—passage in Vero cell culture, C—passage in PEK cell culture.

**Table 3:** Scores in the “Tool Use and Properties” tests battery.

Test	Scores
Three Cups	1
Tool Use: Pulling a Cloth With a Treat	2
Tool Properties: Pulling a Thread with a Treat	2
Tool Properties: A Whole and a Cut Thread with Treats	4

**Table 4:** Scores in the “Memory” tests battery.

Test	Scores
Sticker Cup	1
Spatial Memory: 3 Upside Down Cups and 1 Treat	2
Memory and Association: Sticker Cup and a Barrier	4

**Table 5:** Conventions for the “Reaction to a New Object” test.

Before Infection	3 dpi
A1—the first presentation of object A (red cylinder)	B1—the first presentation of the object (blue cone), was offered as a new object after infection, along with the previously familiar object A4 (red cylinder, the fourth presentation of the object A)
A2—the second presentation of object A (red cylinder)	
A3—the third presentation of object A (red cylinder)	

**Table 6:** Antibody levels detected in PRNT_50_ and ELISA to various domains of the TBEV E protein.

Group	dpi	Monkey #	Anti-TBEV NAb, log	Ab to DIII, log	Ab to sE, log	Ab to DI + II, log
TBEV	7	TBE-1	2.35	-	-	-
		TBE-2	4.49	3.98	3.9	3.93
		TBE-3	1.8	-	-	-
	10	TBE-1	3.23	-	-	-
		TBE-2	4.4	4.1	4.07	4.68
		TBE-3	1.84	-	-	-
POWV	7	POW-1	1.3	-	-	-
		POW-2	1.8	-	-	-
		POW-3	-	-	-	-
TBEV+ POWV	7	T + P − 1	1.9	-	-	-
		T + P − 2	1.4	-	-	-
	10	T + P − 1	1.1	-	-	-
		T + P − 2	3.6	3.49	3.32	2.92
Vac + POWV	−1	vP-1	2.40	3.49	3.33	3.42
		vP-2	2.70	3.75	3.3	3.53
	2	vP-1	2.40	-	-	-
		vP-2	2.57	3.85	3.55	4.77
	4	vP-1	2.98	4.02	4.02	4.41
		vP-2	2.31	4,08	3.84	3.6
	7	vP-1	3.04	4.92	4.84	5.58
		vP-2	2.95	5.33	4.95	4.62
	10	vP-1	4.17	4.45	4.6	4.87
		vP-2	5.30	5.55	5.01	7.82

“-”—not detected. “#”—identification number of the monkey.

**Table 7:** Viremia in TBEV- and/or POWV-infected monkeys detected by plaque assay, qRT-PCR and ELISA.

Group	Monkey #	Plaque Assay	TBEV-Ag ELISA	POWV-*q*RT-PCR, log(GCP)/mL	TBEV-*q*RT-PCR, log(GCP)/mL	Plaque Assay	TBEV-Ag ELISA	POWV-*q*RT-PCR, log(GCP)/mL	TBEV-*q*RT-PCR, log(GCP)/mL
		2 dpi				4 dpi			
TBEV	TBE-1	+	−	n.t.	−	−	−	n.t.	5.07
	TBE-2	+	−	n.t.	−	+	+	n.t.	6.83
	TBE-3	+	−	n.t.	5.84	+	−	n.t.	−
POWV	POW-1	+	−	6.06	n.t.	−	−	−	n.t.
	POW-2	+	−	5.26	n.t.	−	−	−	n.t.
	POW-3	+	−	6.63	n.t.	−	−	−	n.t.
POWV + TBEV	T + P − 1	+	−	4.78	−	−	−	−	−
	T + P − 2	+	+	4.66	5.00	+	+	−	−
Vac + POWV	vP-1	−	−	−	n.t.	−	−	−	n.t.
	vP-2	−	−	−	n.t.	−	−	−	n.t.

“+”—positive, “−”—negative, n.t.—not tested. “#”—identification number of the monkey.

**Table 8:** The presence of TBEV and/or POWV in the spleens, lymph nodes and brains of infected monkeys.

Group	TBEV			POWV			TBEV + POWV				Vac + POWV	
Monkey #	TBE-1	TBE-2	TBE-3	POW-1	POW-2	POW-3	T + P − 1		T + P − 2		vP-1	vP-2
Organ	Log (GCP TBEV)/mL			Log (GCP POWV)/mL			Log (GCP TBEV)/mL	Log (GCP POWV)/mL	Log (GCP TBEV)/mL	Log (GCP POWV)/mL	Log (GCP POWV)/mL	
Spleen	4.97	-	-	4.53	-	-	4.07	-	4.4	-	-	-
Lymph node	3.46	-	4.2	4.85	-	-	-	-	-	-	-	-
Brain	-	-	-	-	-	-	-	-	-	-	-	-

“-“—not detected. “#”—identification number of the monkey.

**Table 9:** Differences in the response to familiar and new objects in infected and control groups in the “Reaction to a New Object” test.

Behavioral Rate	Infected Group		Control Group	
	Statistical Validity (Wilcoxon Test (z))	*P*-Value	Statistical Validity (Wilcoxon Test (z))	*P*-Value
Activity (familiar object) A1/A4	2.2013 *	0.0277 *	2.1582 *	0.0309 *
Activity (new object) A4/B1	1.1531	0.2488	3.4077 *	0.0006 *
Concentration (familiar object) A1/A4	1.5724	0.1158	2.9534 *	0.0031 *
Concentration (new object) A4/B1	1.5724	0.1158	3.4077 *	0.0006 *
Latency A1/A4 or B1 (depending on the first preference of the primate, subject to the presence of two objects at once after infection)	1.1531	0.2488	1.9547	0.0506

An asterisk indicates significant differences (z > 1.96, *P* < 0.05). Results deviating from the norm are highlighted in red. A1/A4—the difference between the first presentation of an object before infection and its subsequent presentation after the infection now as the familiar object. A4/B1—the difference between a previously familiar object and a new object presented after infection.

## Data Availability

Data are contained within the article.

## Supplementary Materials

The following supporting information can be downloaded at: https://www.mdpi.com/article/10.3390/vaccines11121754/s1, Figure S1: Testing the specificity of the TBEV (A) and POWV (B) *q*PCR systems. Amount of virus in the mixes used for each graph is marked; Figure S2: “Three Cups” Test; Figure S3: “Tool Use: Pulling Cloth with Treat” Test; Figure S4: “Tool Properties: Pulling the Thread with Treat” Test; Figure S5: “Tool Properties: Whole and Cut Thread with Treat” Test; Figure S6: “Spatial Memory: 3 Upside Down Cups and 1 Treat” Test; Figure S7: «Box» test; Figure S8: Test No. 4 “Reaction to a New Object”; Figure S9: ALP levels in the serum of the study animals before infection, on the 2nd, 4th and 7th day after infection; Figure S10: ALT levels in the serum of the study animals on the 2nd, 4th and 7th day after infection; Figure S11: AST levels in the serum of the study animals before infection, on the 2nd, 4th and 7th day after infection; Figure S12: GGT levels in the serum of the study animals on the 2nd, 4th and 7th day after infection; Figure S13: Amylase levels in the serum of the study animals before infection, on the 2nd, 4th and 7th day after infection; Figure S14: Prothrombin time of the serum of the study animals on the 2nd, 4th and 7th day after infection; Figure S15: The number of lymphocytes, monocytes, segmented and bacillary neutrophils in blood smears of tested animals on the 0, 2nd, 4th and 7th day after infection; Table S1: Primers for RT and *q*RT-PCR; Table S2: Biochemical parameters of the blood of the studied animals.
