# Supplementary material for: Inapparent Tick-Borne Orthoflavivirus Infection in Macaca fascicularis: A Model for Antiviral Drug and Vaccine Research
Source: Vaccines (Basel). 2023 Nov 25;11(12):1754. doi: 10.3390/vaccines11121754 (PMC10747564; doi:10.3390/vaccines11121754)
Supplement: Supplementary file 1 [file vaccines-11-01754-s001.zip › vaccines-2665728-supplementary.pdf]

Supplementary Information

Inapparent Tick-borne Orthoflavivirus Infection in *Macaca fascicularis*: a Model for Antiviral Drug and Vaccine Research

Victoria Illarionova [1,2], Anastasia Rogova[1], Ksenia Tuchynskaya[1], Viktor Volok[1,3], Yulia Rogova[1], Victoria Baryshnikova[4], Yuriy Turchenko[4], Alexander Litov[1,5], Anna Kalyanova[1], Alexandra Siniugina[6], Aydar Ishmukhametov [5,6], and Galina Karganova [1,5,*]

1 FSASI "Chumakov FSC R&D IBP RAS" (Institute of Poliomyelitis), Laboratory of Arbovirus Biology, Moscow 108819, Russia; sue_polio@chumakovs.su
2 Department of Biology, Lomonosov Moscow State University, Leninskie Gory 1 bd. 3, Moscow, 119991 Russia
3 Research Institute for Systems Biology and Medicine (RISBM), Laboratory of Infectious Immunology, Moscow 117246, Russia; info@sysbiomed.ru
4 FSASI "Chumakov FSC R&D IBP RAS" (Institute of Poliomyelitis), Laboratory of Biochemistry, Moscow 108819, Russia; sue_polio@chumakovs.su
5 Institute of Translational Medicine and Biotechnology, Sechenov First Moscow State Medical University, Moscow 119991, Russia; rektorat@sechenov.ru
6 FSASI "Chumakov FSC R&D IBP RAS" (Institute of Poliomyelitis) Moscow 108819, Russia; sue_polio@chumakovs.su
* Correspondence: karganova@bk.ru

Table S1. Primers for RT and *q*RT-PCR.

Method	Viruses	Primers
RT	TBEV	GTB1R: 5′-CCATTCCGGCTCTGAACTTG-3
	POWV	PVR1: 5′-CGAACGTGATCCTGAGTGTT-3′
	Poliovirus	Pow_681r: 5′-AGACCTTTTCCCCCTAGAT-3′
*q*RT-PCR (direct, reverse, probe)	TBEV	TBEL-1: 5′-TCTGAGGGAGACACACTTGG-3′,<br>TBER-1: 5′-GTGCGCCTGTAAACAAAGAA-3′<br>TBEP-1:<br>5′-(FAM)-TCCTTGGTGCAGCTGTTCAGCC-(BHQ1)-3′
	POWV	PowtestR2: 5′-CGTGACGCAAGAGTAGGTGA-3′<br>PowTestF: 5′-CCTTCACATGAGAGGGCGTC-3′<br>PowTestProbe:<br>5′-(R6G)GCGGGCCAGTGGAAGGGACGC(BHQ2)-3′
	Poliovirus	PVR1: 5′-CGAACGTGATCCTGAGTGTT-3′<br>PVL1: 5′-GGCAGACGAGAAATACCCAT-3′<br>PVP1:5′-(R6G*)-TTGATTCATGAATTTCCTTCATTGGCA-(BHQ1)-3′

Table S2. Biochemical parameters of the blood of the studied animals.

Monkey #	D.p.i.	*GLU (mmol/L)	ALT (U/L)	AST (U/L)	ALP (U/L)	TP (g/L)	ALB (g/L)	BUN (mmol/L)	BILT (umol/L)	PHOS (mmol/L)	CA (mmol/L)	TRIGL (mmol/L)	CHO (mmol/L)	GGT (U/l)	AMYL (U/L)	AMY-P (U/L)	IRON (umol/L)
**TBE-3**	0	7,68			1940,8		31,72	3,11		1,57	2,45	1,4	4,59		372,5	288,5	19
	2	3,86	17		2037,2	75,6	35,3	4,22		0,91	2,37	0,6	3,8	132	366,1	294,2	22,8
	4	5,58	15,8	81,1	1811,4	68,7	36,2	6,03	2,4	0,85	2,46	0,29	3,37	111,5	408,7	315,9	17
	7	3,06	32,2		1413,4	72,4	35,46	4,97		0,79	2,4	0,29	3,84	98,2	398,3	312	293,6
**TBE-1**	0	4,9		40,5	406,4		33,2	3,06	2,4	1,27	2,69	1,5	6,5		217,7	151,1	21,5
	2	3,51	35,2		392	80,3	36,05	2,95		1,42	2,46	1,29	5,63	119,6	197,7	147,4	19,6
	4	3,93	25,8	55,4	338,6	73,1	35,45	3,49	4,3	0,99	2,41	0,76	4,74	107,1	205,1	143,8	10,8
	7	2,71	24,2	60,1	333,3	77,5	35,05	2,98	3,9	0,76	2,51	0,86	4,49	107	156	108,2	19,9
**TBE-2**	0	6,34		30,7	108,7		36,08	3,13	4,6	1,64	2,46	1,5	4,78		336,7	242,9	18,5
	2	4,86	24,8	23,6	89,5	81,2	38,53	3,32	7,2	0,99	2,34	0,43	3,48	36,1	351,4	254	9,9
	4	3,8	30,1	23,7	75,7	81,2	37,67	3,79	4,6	1,35	2,44	0,66	2,95	36,9	383,6	294,8	20,6
	7	3,64	24	27,9	74,5	88,1	37,51	3,13	3,2	1,19	2,45	0,82	3,12	37,3	358,6	313,5	9,9
**POW-2**	0	8,72			146,8		33,93	4,85		1,93	2,44	2,38	6,2		376,2	249	25,9
	2	4,2	22,7		141,3	82,2	38,1	5,01	1,3	1,28	2,43	1,12	5,63	47,9	306	213,9	21,8
	4	4,18	20,1	34,4	117,3	78,5	37,99	5,62	5	1,3	2,46	0,89	5,07	47,3	309,9	222,8	18,5
	7	4,59	24,2		124,5		41,35	6,43	2	1,63	2,75	1,42	5,53	60,9	357,1	248,1	24,7
**POW-3**	0	6,67			308,5		17,58	3,97		1,39	2,07	0,67	2,15		704,5	350,5	5,6
	2	3,64	13,6	27,1	254,3	73,1	20,56	2,96	3,9	0,5	2,04	0,68	2,13	36,2	483,5	246	17,4
	4	3,86	14,4	21,5	192,4	73,2	21,35	3,9	2,8	0,74	2,11	0,52	2,37	37,5	559,9	287,7	2,11
	7	4,38	14,8	40,4	196,8	80	21,47	2,97	0,6	0,62	2,06	0,95	2,49	38,3	625,1	287,4	11,7
**vP1**	*-14*	*5,56*		*61,2*	*242,1*	*79,9*	*29,71*	*3,72*	*1,3*	*1,12*	*2,81*	*0,52*	*6,05*		*474,7*	*362,6*	*24,2*
	0	7,99	28,5	48,6	443,3		39,7	5,68	3,2	1,51	2,53	0,67	8,9	72,8	437,1	294,4	20,3
	2	5,42	18,1	34,9	342	71	34,08	2,3	4,3	1,04	2,3	0,6	5,38	76,5	328	265,8	15
	4	6,69	21,3	49,6	300,4	73	34,78	3,67	2,4	1,1	2,33	0,53	5,1	68,5	382,9	332,3	15,3
	7	5,94	19,9	32	273,9	81,6	35,68	1,76	2,8	1,04	2,41	0,6	5,5	70,4	376	313,5	9,9
**vP2**	*-14*	*6,14*			*328,2*	*84,6*	*36,97*	*5,35*		*1,61*	*2,61*	*1,65*	*7,39*		*465,4*	*334,1*	*22*
	0	8,84	18,7	38,4	352		33,14	3,11	3,9	1,3	2,38	0,53	5,58	80,7	371,7	295,2	15,7
	2	4,93	41,8		377,1	79,7	41,05	4,25		1,54	2,27	0,63	7,9	78,6	352,6	240,8	25,6
	4	7,81	35,5	37,3	325,2	78,9	43,39	4,6	2,4	1,16	2,48	0,34	9,19	70,1	363	272	-
	7	7,07	34,4	39,8	329,9	82,3	42,03	5,69	3,5	1,23	2,55	1,22	8,68	69,6	414,9	303,5	20,3

**T+P-2**	0	3,31		47,9	234,6		35,62	3,91	3,2	1	2,55	0,66	2,91		397,4	287,8	7
	2	3,47	43,2		258,3	78,8	36,14	2,77	1,3	0,94	2,3	0,36	3,11	76,8	335,9	253,5	7,6
	4	4,09	33,4	31,6	214,8	76,5	34,79	4,12	1,7	0,82	2,3	0,36	3,02	70,4	330	252,9	22,5
	7	4,05	52,1	43,5	211,4	80	33,91	3,2	2,8	0,91	2,22	0,39	2,89	66,5	341,8	249,6	22,1
**T+P-1**	0	5,87		39,9	91,2		35,98	5,36	2	1,7	2,59	1	4,15		516,9	310,7	22,8
	2	4,97	36,9	31,5	90,8	78,4	41,67	6,13	2,8	1,17	2,52	0,47	5,26	66,2	430,6	271,7	28,2
	4	5,89	34,9	35,3	75,3	74,8	40,71	7,54	2,4	1,2	2,34	0,5	5,35	60,4	358,8	222,8	13,6
	7	4,35	33,9	62,6	73,2	79,2	40,89	4,86	1,7	1,08	2,51	0,43	5,54	61,5	419,4	252,3	9,5
**POW-1**	0	6,5		53,7	273,5		33,26	3,5	0,9	1,9	2,38	0,65	5,56		973,6	645,8	24,3
	2	4,87	12,3	36	337,5	72,4	36,49	2,51	5,4	1,49	2,3	1,05	5,37	103,8	686,5	462,3	21,9
	4	6,41	14,9	36,1	277,7	72,2	36,94	2,44	4,6	1,46	2,3	0,82	5,26	101,7	631,4	481,1	12,9
	7	5,1	14,7	39,4	267,8	82,9	39,79	2,87	4,3	1,43	2,48	0,53	3,84	46,9	792,4	635,8	17,1
**Control**	0	6,39		52,8	2886,8		34,17	4,76	2	2,04	1,88	1,42	4,24		664,5	475,2	23,7
	2	4,16	20,7	48,8	3006,6	76,9	39,8	4,64	0,9	1,93	1,78	0,77	5	129,1	589	455,7	17
	4	4,03	23,8	53,7	2559,5	73,2	40,48	5,47	2,8	1,82	1,69	0,67	5,18	131,2	518,8	390,4	16,5
	7	3,65	25	61,1	2568,6	79,6	38,74	4,98	2	1,5	1,71	0,72	5,05	123,9	528,4	376,9	17,9
**Control**	0	4,3		43,3	468,2		33,31	5,16	2,8	1,2	2,6	1,26	5,12		412,2	352,3	25,9
	2	4,53	29,2	32,4	517,4	74,1	36,57	5,92	1,7	0,95	2,38	0,91	3,89	47,8	387,2	341,4	13,4
	4	4,4	22,9	24,9	409,8	72,2	36,97	5,07	2,8	0,91	2,39	0,47	3,72	46,8	408	345,4	13,8
	7	5,61	23	22,1	424,6	82,1	37,75	6,14	1,7	0,62	2,51	0,72	3,84	46,9	360,6	293	17,1

*Biochemical analysis of the blood sera was carried out using an automated biochemical analyzer Cobas C111 (Roche, Switzerland) calibrated for alanine aminotransferase (ALT), aspartate aminotransferase (AST), glucose (GLU), alkaline phosphatase (ALP), total protein (TP), albumin (ALB), blood urea (BUN), bilirubin total (BILT), lactate dehydrogenase (LDH), phosphate (PHOS), calcium (CA), triglycerides (TRIGL), cholesterol (CHO), gamma-glutamyl transferase (GGT), amylase (AMYL), pancreatic amylase (AMY-P), and iron (IRON).

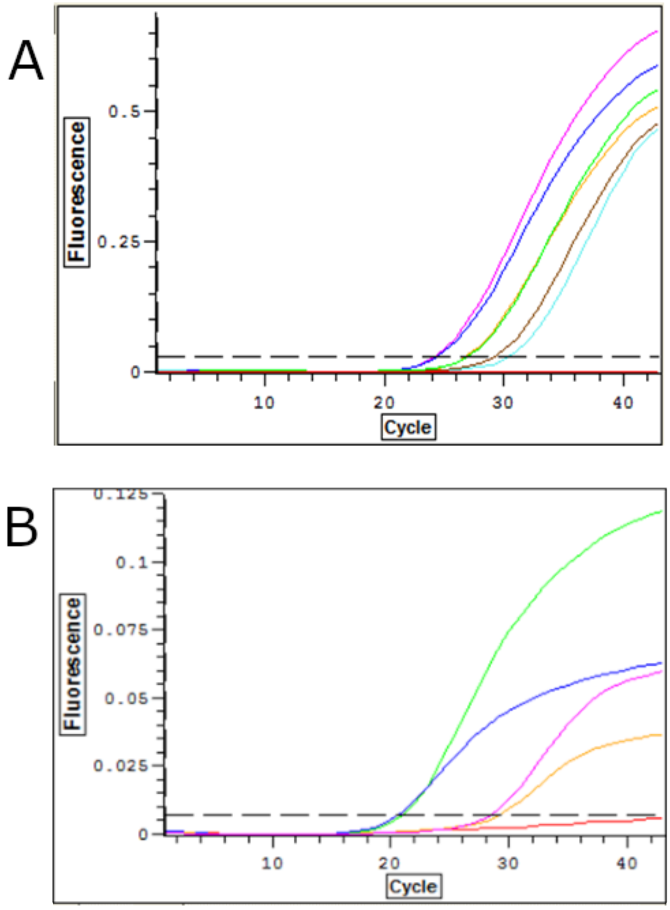

Figure S1. Testing the specificity of the TBEV (A) and POWV (B) *q*PCR systems. Amount of virus in the mixes used for each graph is marked.

Behavioral and cognitive tests: photo material

Battery of Tests No. 1 "Tool Use and Properties"

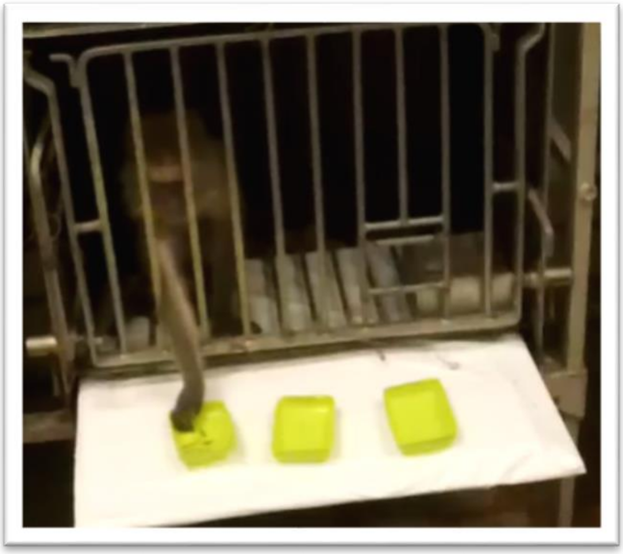

Figure S2. "Three Cups" Test

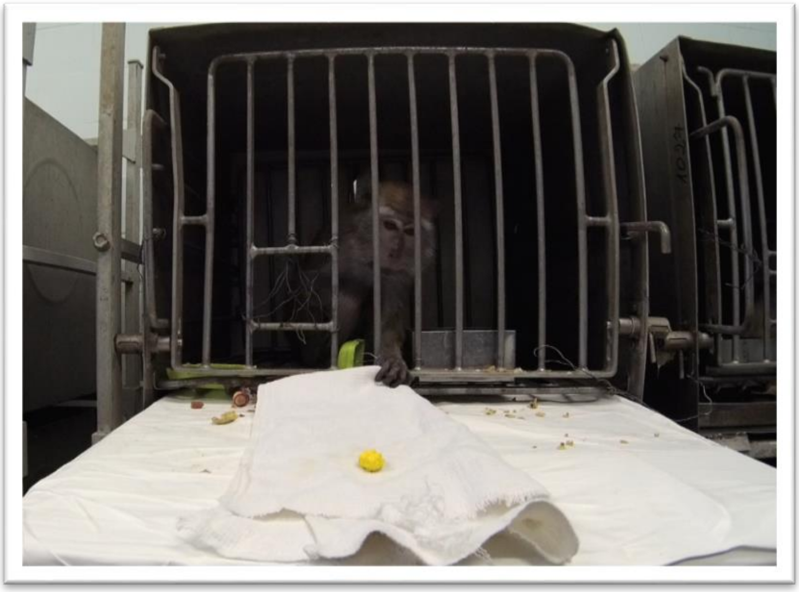

Figure S3. "Tool Use: Pulling Cloth with Treat" Test

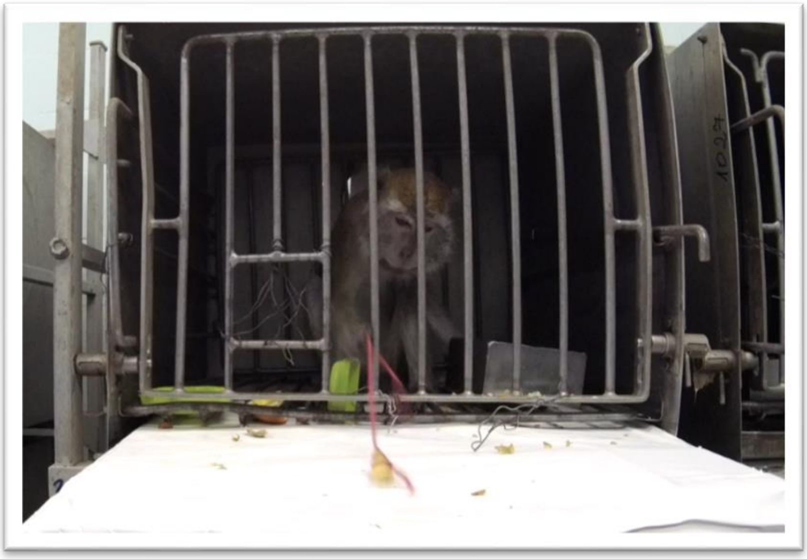

Figure S4. "Tool Properties: Pulling the Thread with Treat" Test

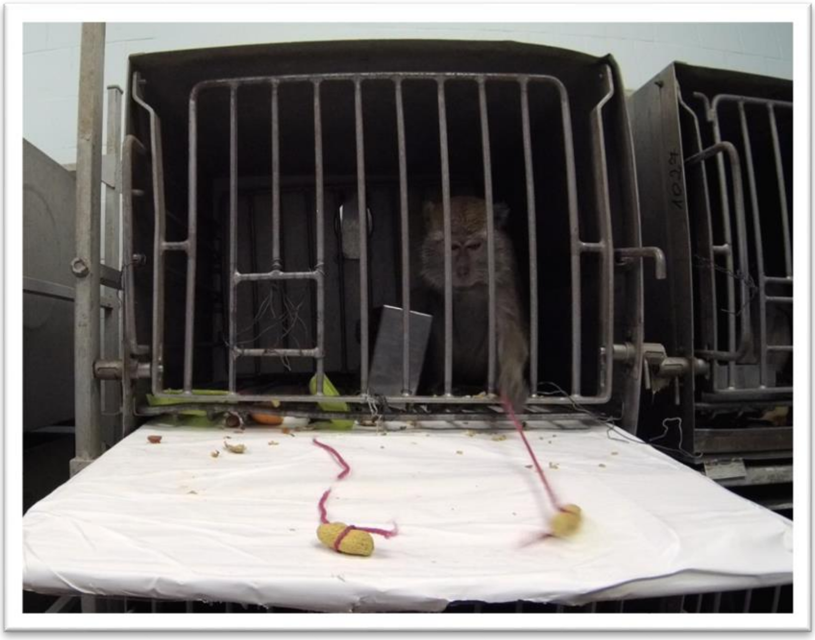

Figure S5. "Tool Properties: Whole and Cut Thread with Treat" Test

Battery of Tests No. 2 "Memory"

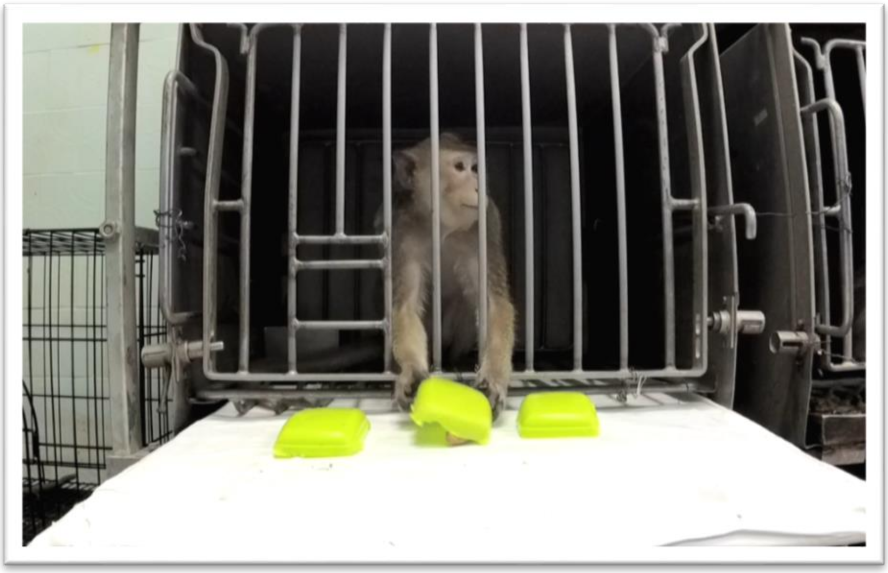

Figure S6. "Spatial Memory: 3 Upside Down Cups and 1 Treat" Test

Test No. 3 "Box"

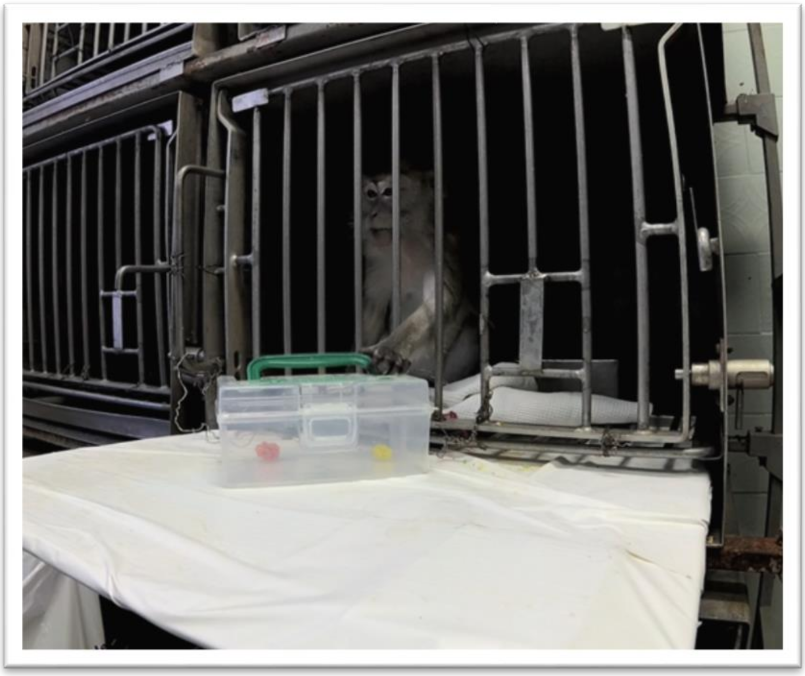

Figure S7. «Box» test.

Test No. 4 "Reaction to a New Object"

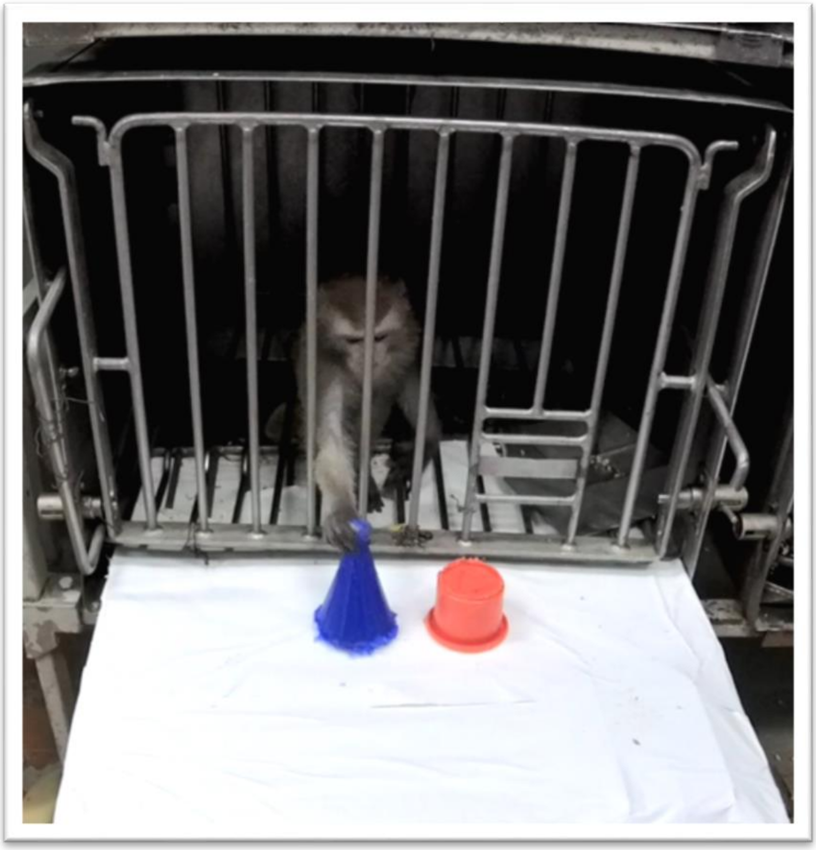

Figure S8. Test No. 4 "Reaction to a New Object"

Serum biochemistry parameters

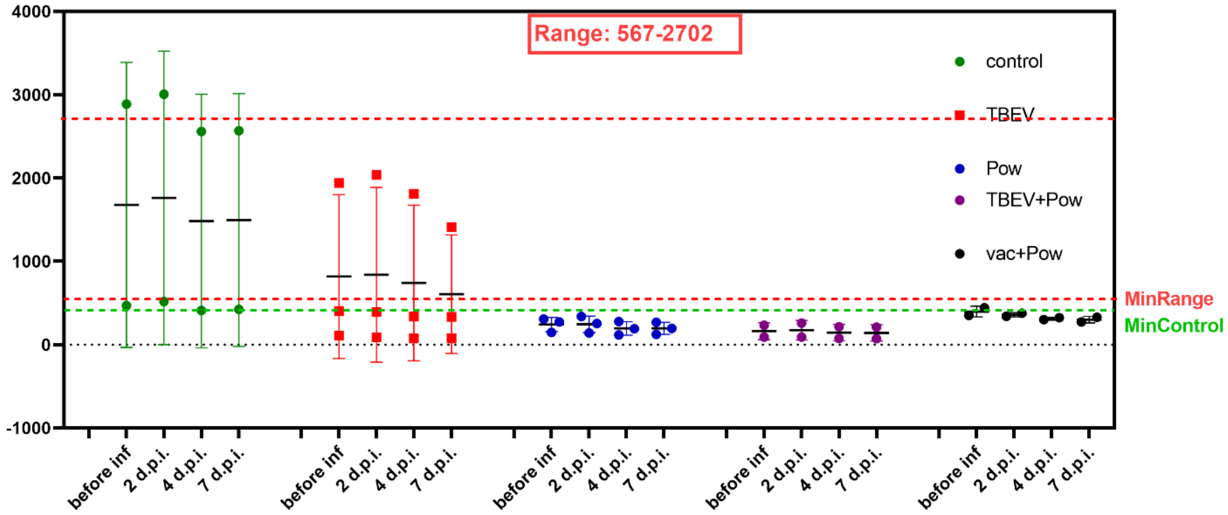

Figure S9. ALP levels in the serum of the study animals before infection, on the 2nd, 4th and 7th day after infection. The red dashed line indicates the minimum and maximum values of this index for *Macaca fascicularis* taken from the article [1]. The green dashed line indicates the minimum and maximum relative to two control monkeys from our experiment.

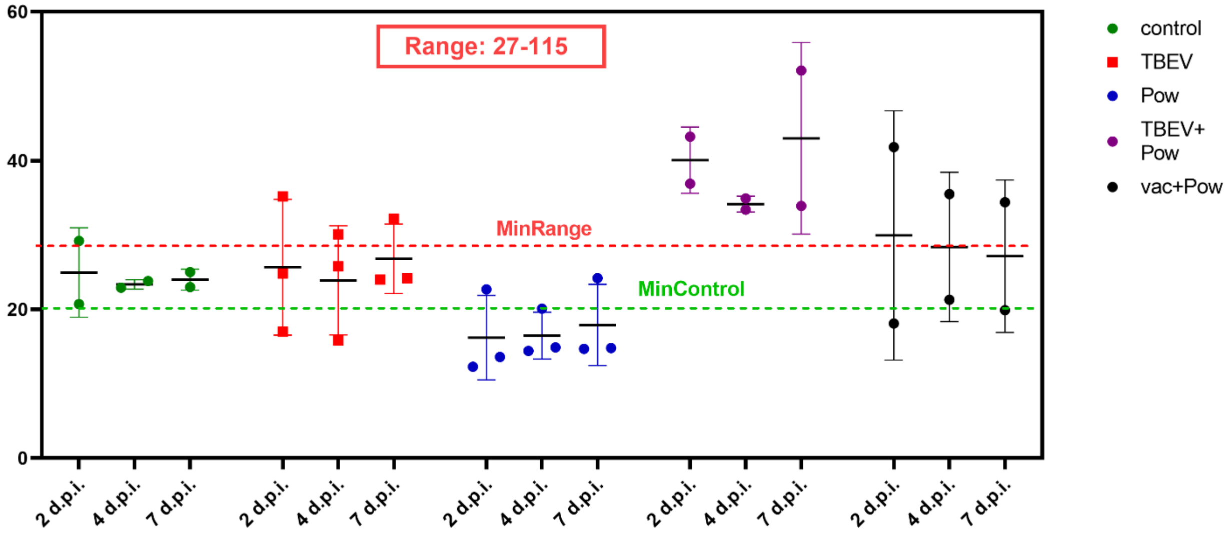

Figure S10. ALT levels in the serum of the study animals on the 2nd, 4th and 7th day after infection. The red dashed line indicates the minimum and maximum values of this index for *Macaca fascicularis* taken from the article [1]. The green dashed line indicates the minimum and maximum relative to two control monkeys from our experiment.

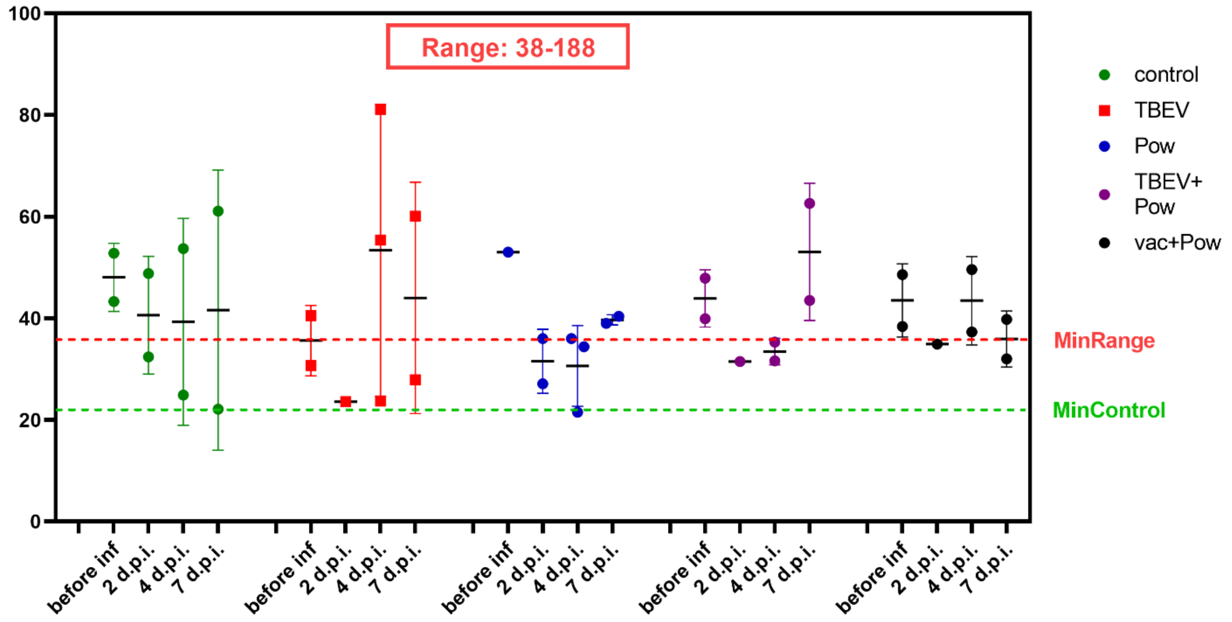

Figure S11. AST levels in the serum of the study animals before infection, on the 2nd, 4th and 7th day after infection. The red dashed line indicates the minimum and maximum values of this index for *Macaca fascicularis* taken from the article [1]. The green dashed line indicates the minimum and maximum relative to two control monkeys from our experiment.

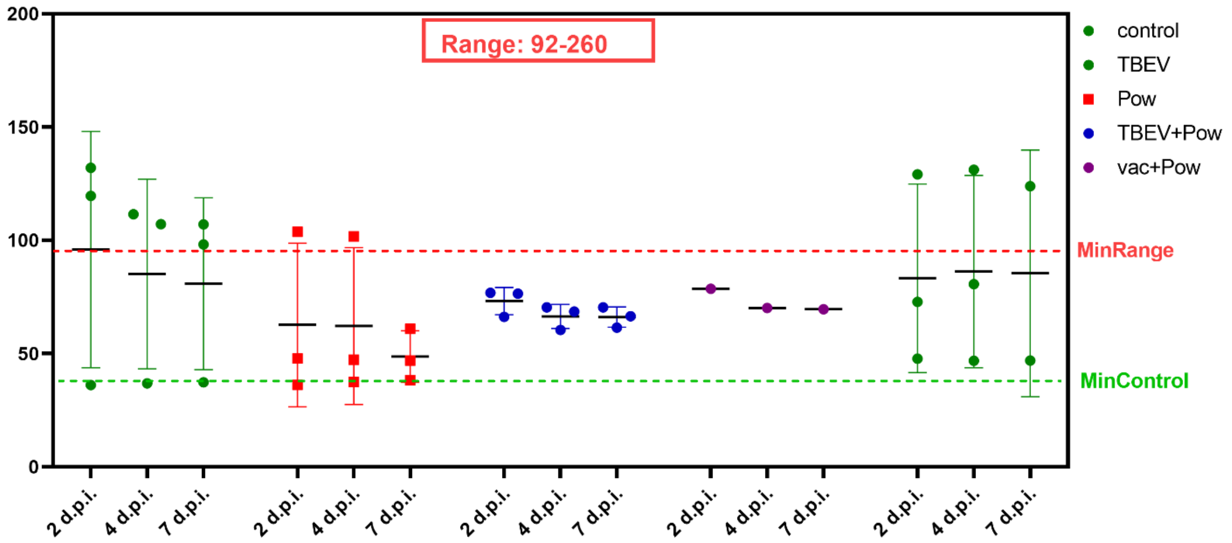

Figure S12. GGT levels in the serum of the study animals on the 2nd, 4th and 7th day after infection. The red dashed line indicates the minimum and maximum values of this index for *Macaca fascicularis* taken from the article [1]. The green dashed line indicates the minimum and maximum relative to two control monkeys from our experiment.

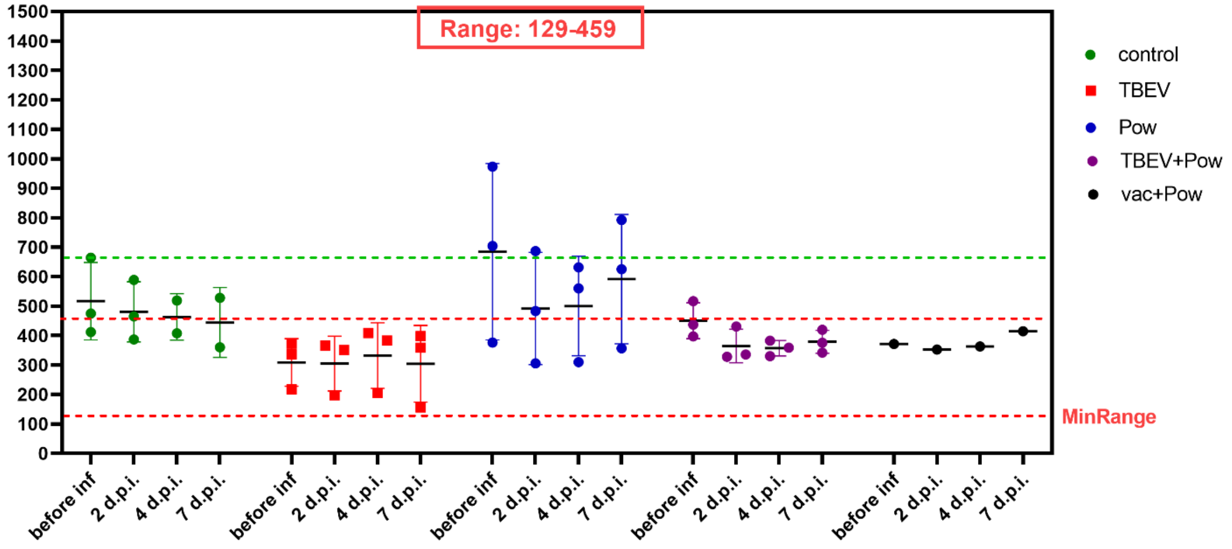

Figure S13. Amylase levels in the serum of the study animals before infection, on the 2nd, 4th and 7th day after infection. The red dashed line indicates the minimum and maximum values of this index for *Macaca fascicularis* taken from the article [1]. The green dashed line indicates the minimum and maximum relative to two control monkeys from our experiment.

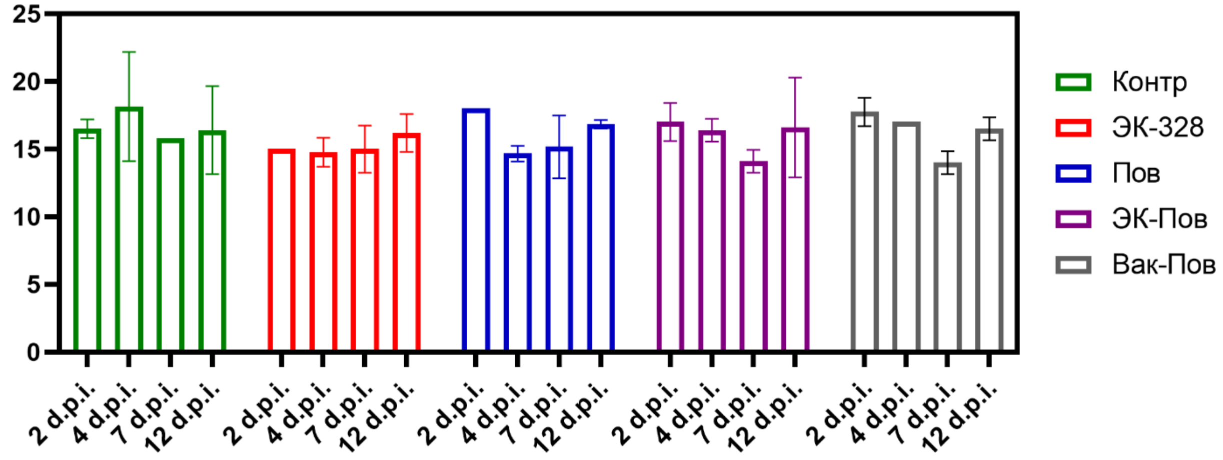

Figure S14. Prothrombin time of the serum of the study animals on the 2nd, 4th and 7th day after infection.

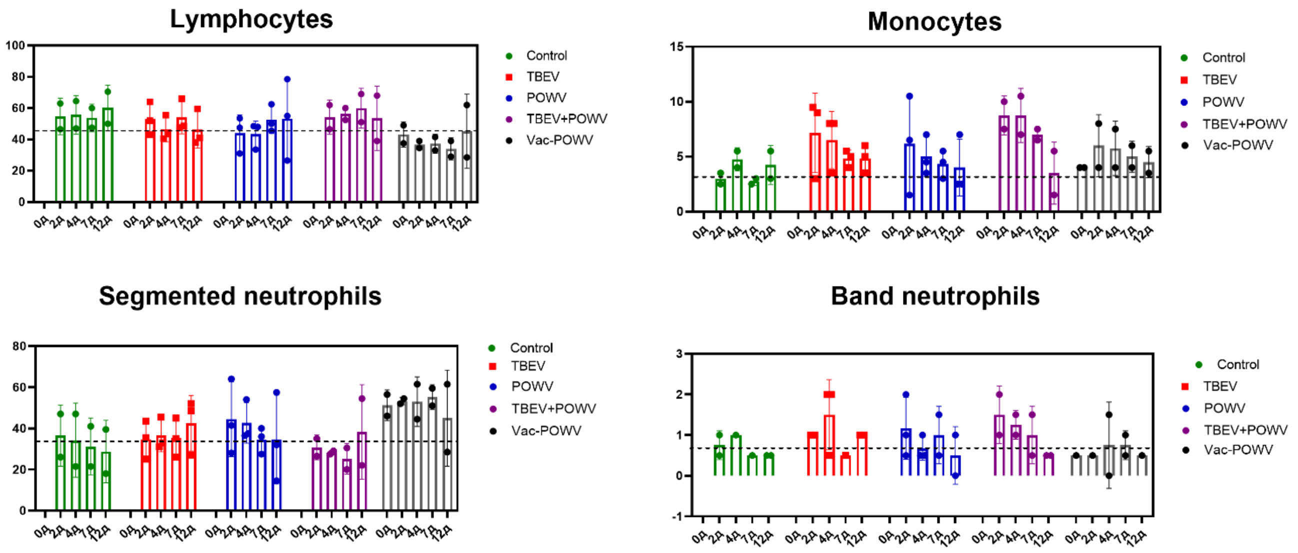

Figure S15. The number of lymphocytes, monocytes, segmented and bacillary neutrophils in blood smears of tested animals on the 0, 2nd, 4th and 7th day after infection.
